# X-Ray microtomography for ant taxonomy: An exploration and case study with two new *Terataner* (Hymenoptera, Formicidae, Myrmicinae) species from Madagascar

**DOI:** 10.1371/journal.pone.0172641

**Published:** 2017-03-22

**Authors:** Francisco Hita Garcia, Georg Fischer, Cong Liu, Tracy L. Audisio, Gary D. Alpert, Brian L. Fisher, Evan P. Economo

**Affiliations:** 1 Biodiversity and Biocomplexity Unit, Okinawa Institute of Science and Technology Graduate University, Onna-son, Okinawa, Japan; 2 Entomology Department, Museum of Comparative Zoology, Harvard University, Cambridge, Massachusetts, United States of America; 3 Entomology Department, California Academy of Sciences, San Francisco, California, United States of America; Institute of Botany, CHINA

## Abstract

We explore the potential of x-ray micro computed tomography (μCT) for the field of ant taxonomy by using it to enhance the descriptions of two remarkable new species of the ant genus *Terataner*: *T*. *balrog* **sp. n.** and *T*. *nymeria* **sp. n.**. We provide an illustrated worker-based species identification key for all species found on Madagascar, as well as detailed taxonomic descriptions, which include diagnoses, discussions, measurements, natural history data, high-quality montage images and distribution maps for both new species. In addition to conventional morphological examination, we have used virtual reconstructions based on volumetric μCT scanning data for the species descriptions. We also include 3D PDFs, still images of virtual reconstructions, and 3D rotation videos for both holotype workers and one paratype queen. The complete μCT datasets have been made available online (Dryad, https://datadryad.org) and represent the first cybertypes in ants (and insects). We discuss the potential of μCT scanning and critically assess the usefulness of cybertypes for ant taxonomy.

## Introduction

The discipline of taxonomy has seen a series of major developments in the last two decades that have transformed the traditional morphology-based approach based on written descriptions and line drawings. Perhaps the most significant change is the shift from pure application of morphology to a complementary mixture of various types of data, such as DNA barcoding [1, 2], multi-locus or whole genome molecular phylogenetic analyses [3–5], morphometrics [6, 7], or ecological niche modelling [8, 9]. Another major development is the rise of a cybertaxonomic infrastructure intended to assist and accelerate the discovery, exploration, classification, and description of new species [10–12]. This infrastructure includes global name-based registers (ZooBank - http://www.zoobank.org), biogeographic databases (AntMaps - http://antmaps.org), genetic sequence databases (GenBank - http://www.ncbi.nlm.nih.gov/nucleotide), digital online libraries (AntBib - http://antcat.org/references); online curation platforms (AntWeb - https://www.antweb.org), virtual identification resources (Pacific Invasive Ants Key - http://idtools.org/id/ants/pia), and virtual platforms for the presentation of biodiversity (Scratchpads - http://www.scratchpads.eu).

Furthermore, the way new species are presented visually in taxonomic publications has been transformed. As pointed out by Akkari et al. [13], modern alpha taxonomy depends heavily upon illustrations in order to communicate and express morphological information in species descriptions. For most of the last two centuries line drawings [14, 15] were the most common method to illustrate characters or specimen morphology. The problem with the use of line drawings in most of the older literature is the schematic nature of the drawings, which, while being adequate for the delineation of species at the time of description, rarely provide enough diagnostic details for a precise and reliable species determination after more taxa are discovered. In addition, most line drawings usually provide a simplified interpretation of morphological characters or whole specimens, which is highly subjective and often not transparent. In the late 20^th^ century scanning electron microscopy (SEM) was sometimes used to illustrate whole specimens within larger revisions or identification guides [16, 17]. The latter technique can provide accurate and detailed visual representations of microscopic structures and characters not easily recognisable with standard light microscopes. Despite being sometimes sufficient for species-level diagnostics [18], the use of SEM for whole specimen imaging often does not provide the desired detail richness and resolution necessary to enhance species descriptions or enable species identifications [17, 19]. In the last decade, this situation changed rapidly with the advent of focal stack imaging, which allowed high-quality two-dimensional photographs, initially in black and white [20, 21] and later in colour [22, 23]. Until the present day, montage photography has continued to be the back-bone of ant taxonomy illustrations for superbly detailed character images, such as male genitalia [24], as well as the presentation of whole specimen morphology and illustrated identification keys [5, 25].

Recent advances in modern computational and microscopic technology have opened new horizons for interactive and three-dimensional (3D) imagery, such as rotational SEM, magnetic resonance imaging, optical projection tomography, confocal laser scanning microscopy, nano x-ray computed tomography (nanoCT), micro x-ray computed tomography (μCT), and synchrotron x-ray tomography [12, 26–30]. These techniques have the potential to revolutionize collections-based studies by allowing detailed assessments of both external and internal morphology of rare and important material without causing damage to specimens. Non-invasive imaging techniques like μCT increase the amount of data that can be extracted from specimens by digitizing entire object volumes. The projection images captured can be reconstructed into volume elements called voxels. The resulting voxel-based datasets can be used to generate volumetric models of specimens that can be both visualized and manipulated in three dimensions with micrometre to nanometre resolution. The virtual reconstructions can be rotated, sectioned, and measured to gain a comprehensive knowledge of the anatomy and morphology of a specimen. An increasing number of studies in systematics and taxonomy have employed volumetric datasets to reconstruct fossils [31–33], study functional morphology [34–36], and discover new phylogenetically important anatomical characters [37]. Volumetric data has also been applied in large-scale systematic analyses to collect morphological data for dozens to hundreds of specimens [38, 39]. Furthermore, μCT data has been used recently in invertebrate taxonomy to enable or enhance the taxonomic descriptions of new species of myriapods [13, 40], spiders [41], flat worms [42, 43], and ants [44, 45].

Data deposition has become increasingly important in providing researchers access to volumetric datasets and associated metadata for specimens. Digital libraries (e.g. Digital Fish Library- http://www.digitalfishlibrary.org) were established to allow users to explore and interact with 3D data. These libraries are useful for education and outreach, yet they only provide access to the processed data in the form of images and animations. Next generation repositories (e.g. Dryad Data Repository; GigaScience Database) provide open access to raw data and also assign a citable digital object identifier (DOI). With a DOI, the digitally accessioned data is akin to a vouchered specimen in a museum, as discussed by Carbayo & Lenihan [43].

A major limitation in systematic research is the accessibility of reference or type material. Natural history collections are often underutilized due to the challenges in obtaining and examining material [10, 46, 47]. Quite often it is problematic or even impossible to gain access to important type material, and visiting each collection that might harbour types is a logistical, time-consuming, and expensive endeavour. The establishment of virtual collections could help overcome these challenges by providing rapid access to accurate representations of type material [12, 30]. The volumetric datasets generated using 3D imaging techniques can be curated in the form of a “cybertype” to provide a permanent digital representation of a species. These cybertypes can be easily and rapidly accessed by using digital platforms without the constraints of traditional specimen loans. Based on the idea of Godfray [10] to "create a new form of type specimen to be displayed on the web using the very best current imaging methods", Faulwetter et al. [12] proposed that datasets intended to serve as a cybertype should fulfil three basic requirements: (a) cybertypes should provide morphological and anatomical information of the same accuracy and reliability as provided by the physical type material; (b) cybertypes should be linked to the original type material; and (3) cybertypes must be retrievable and freely accessible.

Despite the challenges in satisfying these requirements [30], in recent years two new species have been described with a successful designation of cybertypes [13, 40]. Stoev et al. [40] provided the description of a new centipede based on an integrative approach combining morphology, DNA barcoding, transcriptomics, a video of the living specimen, and μCT scans with the combination of all of these datasets representing the first ever published cybertype. This approach is likely the most complete species description ever undertaken for a new species of invertebrate. Recently, Akkari et al. [13] described a new millipede, in which they use μCT data as significant part of the morphological examination and description of the new species. The cybertypes of the holotype and one paratype consist of the entire raw scanning data of all body parts. Both studies fit the cybertype criteria postulated by Faulwetter et al. [12], namely anatomical fidelity, association with physical type material, and open accessibility. Surprisingly, apart from these two myriapod descriptions, no other cybertypes of new species have been designated so far.

The myrmicine ant genus *Terataner* Emery currently holds 12 valid species, which are equally distributed in the Afrotropical and Malagasy zoogeographical regions [48]. *Terataner* are very conspicuous, heavily armoured, medium-sized to large arboreal or sub-arboreal ants that prey on ants, termites and other insects [49]. Bolton [50] provided the first modern taxonomic analysis of *Terataner*, in which he diagnosed the genus, re-described all Afrotropical species, described one new Malagasy species, gave species accounts for the remainder, and presented identification keys for all species. The latter study represents a solid foundation for the taxonomy of the genus, but no subsequent taxonomic studies have been published. Nevertheless, most modern ant inventories in the Afrotropics [51, 52, 53] and, more importantly, in the Malagasy region [54–56] were carried out after Bolton's [50] revision. These studies generated 99% of the currently available material of *Terataner*, most of it originating from Madagascar, and to a lesser extent the neighbouring islands of the South West Indian Ocean. First examination of this material revealed that most of the species richness of *Terataner* is actually undescribed with upwards of 40 new Malagasy species. Based on these findings, the genus is in need of further taxonomic treatments.

The currently known species can be divided into two groups with contrasting biological lifestyles [49]. The first, smaller group with just the four species of the *T*. *luteus* complex is restricted in its distribution to the rainforests of West and Central Africa. All four species are strictly arboreal and nest in rotten parts of standing timber at a considerable distance from the ground [50]. The female reproductives are morphologically typical ant queens with wings. The second and much larger group contains the remaining eight valid species (plus most undescribed species) and is predominantly found on Madagascar with few species on the Seychelles, Mayotte, the Comoros, and East and South Africa. They tend to nest subarboreally in the lower vegetation in preformed nest cavities, such as hollow twigs and burrows of wood-boring insects [49]. Intriguingly, the female reproductives of the second group are wingless, ergatoid queens that are often difficult to distinguish from the workers.

In this study, we focus only on the description of two morphologically exceptional new species of *Terataner* that we have separated from a larger-scale revision of the whole genus in order to test and discuss the applicability of μCT for ant taxonomy. Using μCT scanning for 50 species requires considerable resources and is obviously out of scope to test a new methodology. Both species are integrated into the existing taxonomic system by providing diagnoses and an updated species identification key for Madagascar on the basis of the worker caste. In addition, we deliver detailed taxonomic descriptions for both new species including discussions, measurements, natural history data, high-quality montage images and distribution maps. We also present the first description of an ergatoid queen for the genus. We also include 3D surface reconstructions (as 3D PDFs), 2D snapshots of 3D models, and 3D rotation videos for both holotype workers and one paratype queen based on volumetric data generated using μCT. We discuss the use of the new technology for ant taxonomy and provide an outlook of further applications. The complete μCT datasets are available online and represent the first cybertypes designated for any insect taxon. The cybertypes were generated from dry-mounted museum specimens, and we critically assess their usefulness for ant taxonomy on the basis of the criteria suggested by Faulwetter et al. [12].

## Material and methods

### Abbreviations of museum depositories

The collection abbreviations given below follow Evenhuis [57]. The material upon which this study is based on is located and/or was examined at the following institutions:

BMNH        The Natural History Museum, London, U.K.

CASC        California Academy of Sciences, San Francisco, U.S.A.

HLMD        Hessisches Landesmuseum Darmstadt, Darmstadt, Germany

MCZC        Museum of Comparative Zoology, Harvard University, Cambridge, U.S.A.

### Material examined and terminology

**Fig 1:**
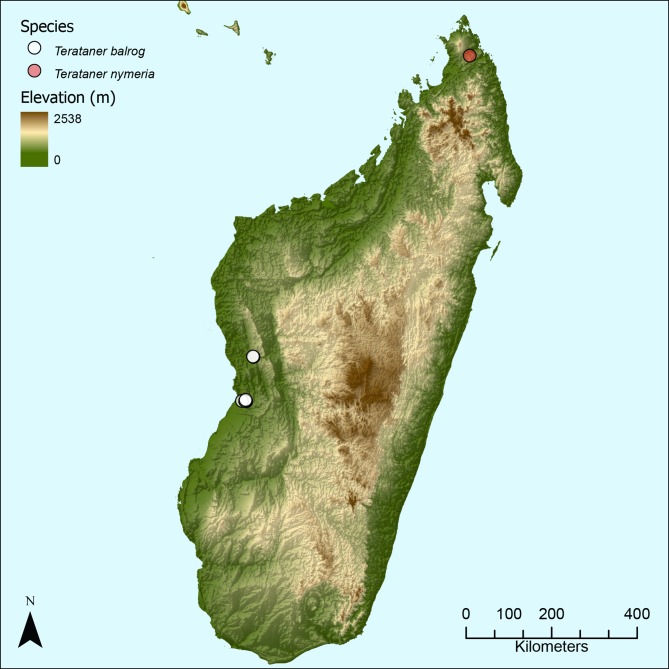
Distribution map. Map of Madagascar showing the known distribution ranges of *T*. *balrog* sp. n. and *T*. *nymeria* sp. n.

The material for this study was collected independently by BLF and GDA during ant inventories carried out in the Malagasy region from 1989 to 2013 (Fig 1). Unless otherwise noted in this publication, it is located in the ant collections of CASC and MCZC. Ant samples used in this study comply with the regulations for export and exchange of research samples outlined in the Convention of Biology Diversity and the Convention on International Trade in Endangered Species of Wild Fauna and Flora. For field work conducted in Madagascar, permits to research, collect and export ants were obtained from the Ministry of Environment and Forest as part of an ongoing collaboration between the California Academy of Sciences and the Ministry of Environment and Forest, Madagascar National Parks and Parc Botanique et Zoologique de Tsimbazaza. Authorization for export was provided by the Director of Natural Resources. Approval Numbers: No. 0142N/EA03/MG02, No. 340N-EV10/MG04, No. 69 du 07/04/06, No. 065N-EA05/MG11, No. 047N-EA05/MG11, No. 083N-A03/MG05, No. 206 MINENVEF/SG/DGEF/DPB/SCBLF, No. 0324N/EA12/MG03, No. 100 l/fEF/SG/DGEF/DADF/SCBF, No. 0379N/EA11/MG02, No. 200N/EA05/MG02. All specimen data are freely accessible on Antweb (http://www.antweb.org). Each specimen used in this study can be traced by a unique specimen identifier affixed to the pin (e.g. CASENT0053630). The general terminology for ant morphology is mostly based on Bolton [16, 50]. The terminology for the description of surface sculpturing follows Harris [58] and Bolton [50] while the terminology for the description of inclination of pilosity follows Wilson [14].

### Measurements and indices

Morphometric measurements of the physical specimens were performed with a Leica M125 microscope equipped with an orthogonal pair of micrometres at magnifications ranging from 63 to 100x. These measurements and indices are presented as minimum and maximum values with arithmetic means in parentheses. All measurements were recorded in mm to three decimal places, but are expressed in the study to two decimal places. Most measurements and indices used in this study are based on Bolton [50], Ward [59], Boudinot & Fisher [60], and Hita Garcia & Fischer [61], while some are used here for the first time. In order to compare the dimensions of the virtual 3D reconstructions with the physical specimens, we also measured all three cybertypes. Virtual measurements were done with the 3D measuring function in Adobe Acrobat Pro DC (version 2015.006.30244). These measurements are recorded and compared with the data from the physical specimens in mm to three decimal places. We use error rate, which is the difference between repeated measures divided by 2, as an indicator of accuracy.

**Fig 2:**
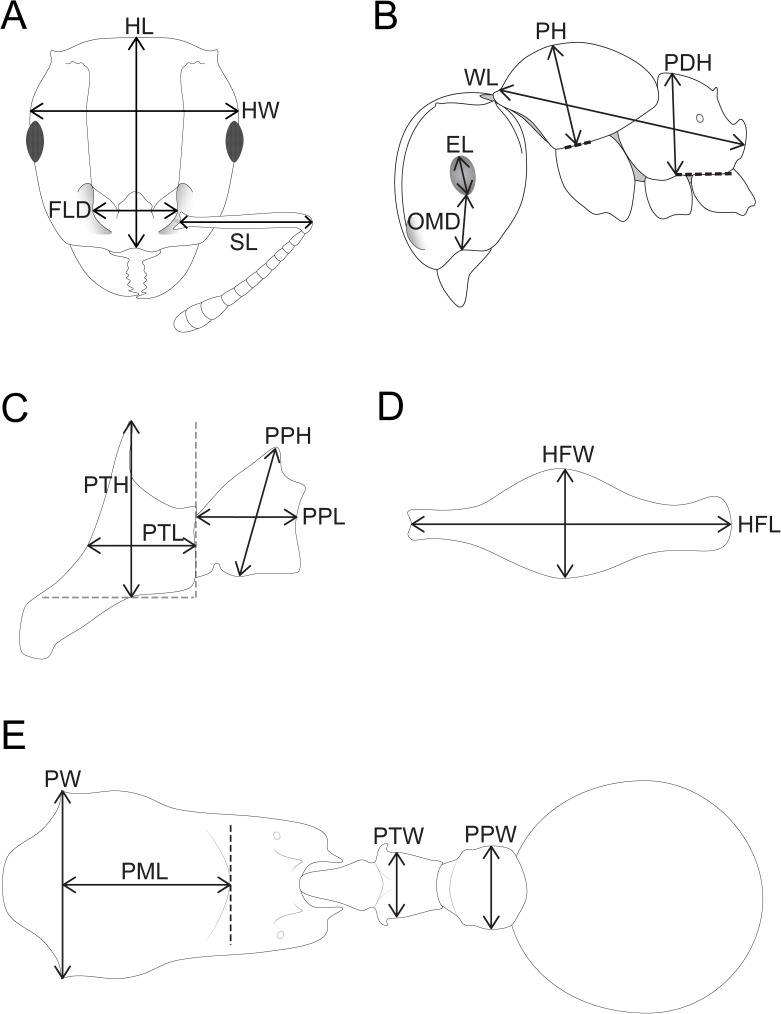
Schematic line drawings illustrating the measurements used in this study. Head in full-face view with measuring lines for HL, HW, FLD, and SL (A), Head and mesosoma in profile with measuring lines for EL, OMD, WL, PH, and PDH (B), Waist segments in profile with measuring lines for PTH, PTL, PPH, and PPL (C), Metafemur in dorsal view with measuring lines for MFL and MFW (D), Mesosoma, waist segments, and gaster in dorsal view with measuring lines for PW, PML, PTW, and PPW (E).

**HL** Head length: maximum distance from the midpoint of the anterior clypeal margin to the midpoint of the posterior margin of head, measured in full-face view. Impressions on the anterior clypeal margin and the posterior head margin reduce head length (Fig 2A).
**HW** Head width: width of the head directly behind the compound eyes, measured in full-face view (Fig 2A).
**FLD** Frontal lobe distance: maximum distance between external borders of frontal lobes, measured in full-face view (Fig 2A).
**SL** Scape length: maximum scape length excluding basal condyle and neck (Fig 2A).
**EL** Eye length: maximum diameter of compound eye, measured in oblique lateral view (Fig 2B).
**OMD** Oculo-malar space: minimum distance between anterior (lower) margin of the compound eye and the mandibular junction, measured in lateral view (Fig 2B).
**WL** Weber's length: in lateral view, maximum diagonal length of mesosoma measured from anterior inflection of pronotum to posterolateral corner of the metapleuron or the metapleural lobes, whichever is most distant (Fig 2B).
**PH** Pronotal height: maximum height of the pronotum measured in lateral view (Fig 2B).
**PDH** Propodeum height: height of the propodeum, measured in lateral view, from the base of the metapleuron to the maximum height of the propodeum, along a line orthogonal to the lower metapleural margin (Fig 2B).
**PTH** Petiolar node height: maximum height of petiolar node, measured in lateral view, from the highest point of the node (**or** any dorsal projection, such as spine, tooth, plate) to the ventral outline. The measuring line is placed at an orthogonal angle to the ventral outline of the node (Fig 2C).
**PTL** Petiolar node length: length of the petiolar node, measured in lateral view, in a line from an orthogonal line projecting from the posterior margin of the node to the angle separating peduncle from node (Fig 2C).
**PPL** Postpetiole length: in lateral view, maximum length of the postpetiole measured from the anterior to posterior inflection (Fig 2C).
**PPH** Postpetiole height: maximum height of the postpetiole, measured in lateral view, from the highest point of the node (or any dorsal projection, such as spine, tooth, plate) to the ventral-most point of sternal process (Fig 2C).
**MFL** Metafemur length: maximum length of metafemur from the distal margin of the trochanter to the metafemur apex, measured in dorsal view (Fig 2D).
**MFW** Metafemur width: maximum width of metafemur, measured in dorsal view (Fig 2D).
**PW** Pronotal width: maximum width of anterior pronotum including the anterolateral spines/horns/teeth/angles, measured in dorsal view (Fig 2E).
**PML** Promesonotal length: length of promesonotum from the measuring line for PW to the posterior margin of the mesonotum, measured in dorsal view (Fig 2E).
**PTW** Petiolar node width: maximum width of petiolar node (**not** the width between dorsal projections, such as spines, teeth, lobes), measured in dorsal view (Fig 2E).
**PPW** Postpetiole width: maximum width of postpetiole, measured in dorsal view (Fig 2E).

**CI** Cephalic index: HW / HL * 100
**FLI** Frontal lobes index: FLD / HW *100
**OI** Ocular index: EL / HW * 100
**OMI** Oculo-Malar index: EL / OMD * 100
**SI** Scape index: SL / HW * 100
**DMI** Dorsal mesosoma index: PW / WL * 100
**LMI** Lateral mesosoma index: PH / WL * 100
**PMI** Promesonotum index: PW / PML * 100
**PPDI** Pronoto-Propodeum index: PH / PDH * 100
**MFLI** Metafemur length index: MFL / HW *100
**MFI** Metafemur index: MFL / MFW * 100
**LPeI** Lateral petiolar node index: PTL / PTH * 100
**DPeI** Dorsal petiolar node index: PTW / PTL * 100
**LPpI** Lateral postpetiole index: PPL / PPH * 100
**DPpI** Dorsal postpetiole index: PPW / PPL * 100

### Montage images and illustrations

The raw images from both new species were taken with a Leica DFC450 camera attached to a Leica M205C microscope and Leica Application Suite (version 4.1). The raw photo stacks were then processed to single montage images with Helicon Focus (version 6). Additional montage images used for the illustrated identification key were taken from AntWeb. These we were either created by using a JVC KY-F75 digital camera and Syncroscopy Auto-Montage software (version 5.0), or a Leica DFC425 camera in combination with the Leica Application Suite software (version 3.8). All montage images used in this publication are available on AntWeb. Vector illustrations were created with Adobe Illustrator (version CS 5) by tracing specimen photographs.

### Micro x-ray computed tomography

Micro x-ray computed tomography (μCT) scans were created using a ZEISS Xradia 510 Versa 3D X-ray microscope and the ZEISS Scout and Scan Control System software (version 10.7.2936). The scanned specimens were left attached to their paper point, which was glued to one end of a 4 cm long MicroLumen high performance polyimide tube. Scan settings were selected according to yield optimum scan quality: 4x objective, exposure times either 2.5 or 3 s, binning of two by two pixels, source filter “Air”, voltage between 45 and 60 keV, power between 3.5 and 5 W, current between 78 to 83 μA, and field mode “normal”. The combination of voltage, power and exposure time was set to yield intensity levels of between 10,000 and 15,000 across the whole specimen. Scanning duration was approximately 2 h, depending on exposure time. Full 360 degree rotations were done with a number of 1601 projections. The resulting scans have resolutions of 1013x992x993 (HxWxD) pixels and voxel sizes range between 4.69 μm and 5.41 μm. Three type specimens were used for μCT scanning. An overview of the specimens used and scanning settings is provided in Table 1.

**Table 1:** Data summary for μCT scanning. Overview of the three scanned Terataner type specimens providing specimen data, file type, scan settings, and voxel sizes for the resulting scans.

Species	Specimen code	Caste	Type status	File type	Voltage (keV)	Amperage (μA)	Power (W)	Exposure (s)	Source distance (mm)	Detector distance (mm)	Voxel size (μm)
*T*. *balrog*	CASENT0472559	worker	holotype	DICOM	60	83	5	3	60	15	5.405
*T*. *balrog*	CASENT0426614	queen	paratype	DICOM	45	78	3.5	2.5	25	11	4.691
*T*. *nymeria*	CASENT0053630	worker	holotype	DICOM	50	80	4	2.5	40	13	5.099

3D reconstruction of the resulting scans was done with XMReconstructor (version 10.7.2936) and saved in DICOM file format (default settings; USHORT 16 bit output data type). These were accessed with Amira software (version 6.0). For rotation videos and still images the Volren function was used to create the 3D rendering, the colour space range adjusted to minimum so that the exterior surface (without pilosity, see discussion below) of the specimen remained visible (shading: user-defined > coefficients ka = 0.15, kd = 0.5, ks = 0.5; light angle: user-defined > light angle yaw = 0.0357, light angle pitch = -0.1726). The rotation videos were created with the Camera Path object (10 keyframes, constant velocity for constant rotation speed) and movie maker functions (parameters: mpeg format, AntiAlias2, total of 1500 frames at 50 frames per second, and resolution of 1280 by 720 pixels).

The first step to creating 3D PDFs was to make 3D renderings of ant specimens in Amira using the Isosurface function (deselect compactify) for exporting surface meshes in the STL file format. These were imported into Meshlab (version 1.3.3) where the number of vertexes per specimen was reduced in three steps to decrease total file size and before importing into Adobe Acrobat. The scan files were cleaned from isolated vertexes (Filters > Cleaning and Repairing > Remove isolated pieces (wrt diameter) [set max diameter: 0.05–1%]) and the paper tips on which the ants are mounted were digitally removed. The next step removed all internal vertexes so that only the exoskeleton remained (1. Filters > Color Creation and Processing > Ambient Occlusion Per Vertex; 2. Filters > Selection > Select Faces By Vertex Quality (min = 0, max = 0.001); 3. Remove Selected Faces). In the last step, the number of total vertexes was reduced to the final number of <1,000,000 (Filters > Remeshing, Simplification and Reconstruction > Quadratic Edge Collapse Decimation) in order to get a manageable resolution resulting in 3D PDF files less than 30 MB in size. They were annotated and exported as 3D PDFs in Adobe Acrobat Pro DC (version 2015.006.30119) using the Tetra4D Converter plug-in (version 5.1.2). When viewing the 3D pdfs with Adobe Acrobat Reader (version 8 or higher), trusting the document by clicking on the image will activate the interactive 3D-mode and allows rotating, moving and zooming into the 3D model. The 3D PDFs and 3D rotation video files are downloadable from the supporting information (see overview Table 2) and the cybertype original volumetric datasets (DICOM files) have been archived at the Dryad Data Repository (http://dx.doi.org/10.5061/dryad.sk6s0).

**Table 2:** Overview of 3D PDF files and rotation videos available in the supporting information.

File name	Species	Specimen code	Type status	Caste	File type
S1 Fig	Terataner balrog	CASENT0472559	holotype	worker	3D PDF
S2 Fig	Terataner balrog	CASENT0426614	paratype	queen	3D PDF
S3 Fig	Terataner nymeria	CASENT0053630	holotype	worker	3D PDF
S1 Video	Terataner balrog	CASENT0472559	holotype	worker	3D rotation video
S2 Video	Terataner balrog	CASENT0426614	paratype	queen	3D rotation video
S3 Video	Terataner nymeria	CASENT0053630	holotype	worker	3D rotation video

### Nomenclatural acts

The electronic edition of this article conforms to the requirements of the amended International Code of Zoological Nomenclature, and hence the new names contained herein are available under that Code from the electronic edition of this article. This published work and the nomenclatural acts it contains have been registered in ZooBank, the online registration system for the ICZN. The ZooBank LSIDs (Life Science Identifiers) can be resolved and the associated information viewed through any standard web browser by appending the LSID to the prefix “http://zoobank.org/”. The LSID for this publication is: urn:lsid:zoobank.org:pub:08D7D026-8952-49C3-92D2-748C1BE4BF70. The electronic edition of this work was published in a journal with an ISSN, and has been archived and is available from the following digital repositories: PubMed Central, LOCKSS.

## Results and discussion

### Biology and ergatoid reproductives

With the relatively limited material available at the time, Alpert [49] observed the presence of ergatoid reproductives in six species collected from Madagascar. Based on the extensive material presently available to us, we confirm that approximately 40 species (described plus undescribed) of Malagasy *Terataner* species produce ergatoid queens. The same is true for *T*. *bottegoi* from East Africa, from which we have two whole colonies available that each contain one ergatoid queen. We do not know the queen morphology of *T*. *transvaalensis* or of a few undescribed Malagasy species due to scarcity of available material, but based on the overall presence of ergatoid reproductives in these groups, we are predict that these species will also have ergatoid queens. Our examination of approximately 4,000 *Terataner* specimens from over 200 localities suggest ergatoid queens are collected only by sampling entire nests from hollow sticks, branches or other plant material. Moreover, it also seems that colonies of *Terataner* are monogynous since all examined colonies contained only one ergatoid reproductive. Nevertheless, the ergatoid queen’s role within the colony, mating behaviour, and colony establishment remain unknown. Considering that the ergatoid is wingless, the latter is thought to happen by colony fission [49]. Observations of living colonies in Madagascar are required to shed light on the biology of these intriguing ants. For the two species treated herein we were able to identify the ergatoid queen caste of one of them, and consequently provide the first formal description of an ergatoid *Terataner* queen.

### Synoptic list of valid *Terataner* species from Madagascar

*Terataner alluaudi* (Emery, 1895) [Eastern and Northern Madagascar]
*Terataner balrog* Hita Garcia **sp. n.** [Northern Madagascar]
*Terataner foreli* (Emery, 1899) [Eastern and Northern Madagascar]
*Terataner nymeria* Hita Garcia **sp. n.** [Western Madagascar]
*Terataner rufipes* Emery, 1912 [Southern and Western Madagascar]
*Terataner steinheili* (Forel, 1895) [Eastern Madagascar]
*Terataner xaltus* Bolton, 1981 [Southern and Western Madagascar]

### Identification key to *Terataner* species from Madagascar

The following key to species only diagnoses the valid species from the island of Madagascar and should be considered preliminary.

**Fig 3:**
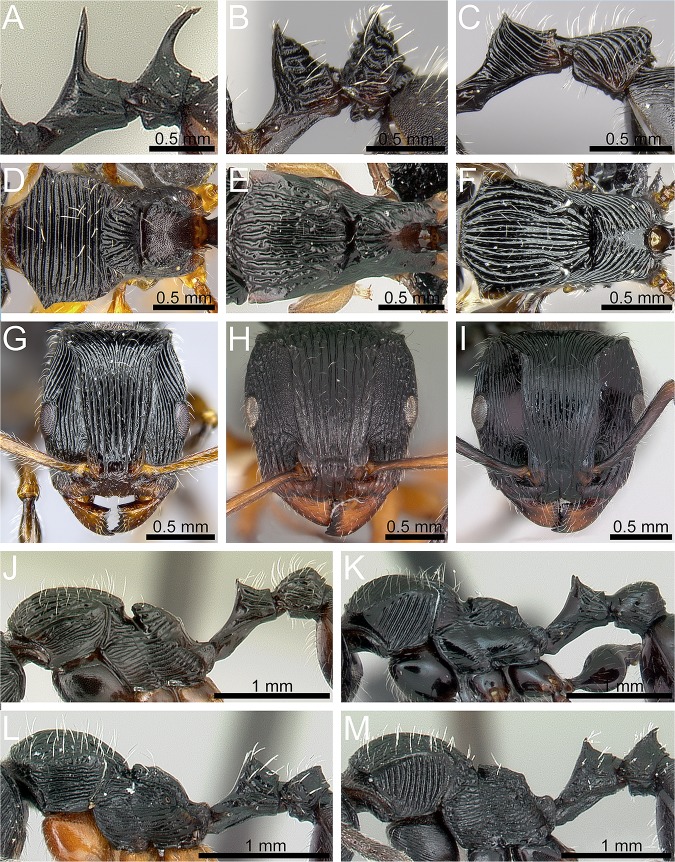
Taxonomic characters used for species diagnostics. Petiole and postpetiole in profile: *T*. *alluaudi* (Emery) (CASENT0095826) (A), *T*. *balrog* **sp. n.** (CASENT0472559) (B), *T*. *nymeria* **sp. n.** (CASENT0053630) (C); mesosoma in dorsal view: *T*. *balrog* (CASENT0472559) (D), *T*. *foreli* (Emery) (CASENT0077299) (E), *T*. *nymeria* (CASENT0053630) (F); head in full-face view: *T*. *nymeria* (CASENT0053630) (G); *T*. *rufipes* Emery (CASENT0102235) (H), *T*. *steinheili* (Forel) (CASENT0172827) (I); mesosoma and waist segments in profile: *T*. *foreli* (CASENT0077299) (J), *T*. *steinheili* (CASENT0172827) (K), *T*. *rufipes* (CASENT0446582) (L), *T*. *xaltus* Bolton (CASENT0002552) (M). Images A, D, G-L reproduced from https://www.antweb.org.

1. Petiole with two very long spines and postpetiole with one very long median spine (Fig 3A). ***T*. *alluaudi***
  - Petiole and postpetiole without very long spines (Fig 3B and 3C). **2**
2. Dorsum of pronotum with conspicuously transverse sculpture (Fig 3D). ***T*. *balrog***
  - Dorsum of pronotum with conspicuously longitudinally arranged sculpture (Fig 3E and 3F). **3**
3. In full-face view head comparatively narrow (CI 87–91) with weakly convex sides, broadest at eye level and conspicuously narrowing towards posterior head margin before broadening again into well-developed angulate posterodorsal corners (Fig 3G). ***T*. *nymeria***
  - In full-face view head never shaped as above (Fig 3H and 3I). **4**
4. In profile postpetiole conspicuously rounded dorsally (Fig 3J and 3K); in dorsal view rounded, not shield-like nor with a transverse crest or ridge. **5**
  - In profile postpetiole cuneiform (Fig 3L and 3M); in dorsal view shield-like with a transverse crest or ridge. **6**
5. Metanotal groove strongly and deeply impressed; sculpture on lateral pronotum longitudinally rugose (Fig 3J). ***T*. *foreli***
  - Metanotal groove distinct but only moderately impressed; sculpture on lateral pronotum diagonally costate (Fig 3K). ***T*. *steinheili***
6. Surface sculpture on lateral pronotum longitudinally rugose; procoxae usually unsculptured, rarely with traces of sculpture; legs yellowish light brown (Fig 3L). ***T*. *rufipes***
  - Surface sculpture on lateral pronotum transversely costate; procoxae conspicuously longitudinally rugulose/rugose; legs dark brown to black (Fig 3M). ***T*. *xaltus***

### *Terataner balrog* Hita Garcia sp. n.

urn:lsid:zoobank.org:act:B191C56B-B7A5-4596-A4C0-F15AB05FEC13

(Figs 1, 3B, 3D and 4–7, S1 and S2 Figs, S1 and S2 Videos)

**Fig 4:**
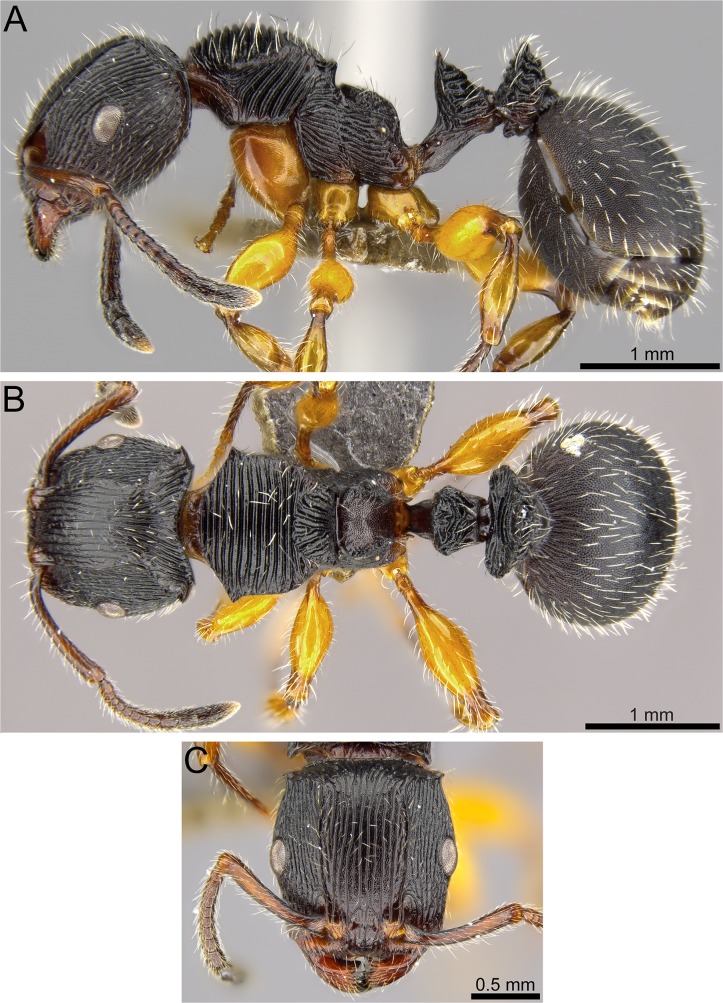
*Terataner balrog* sp. n. holotype worker (CASENT0472559). Body in profile (A), body in dorsal view (B), head in full-face view (C).

**Fig 5:**
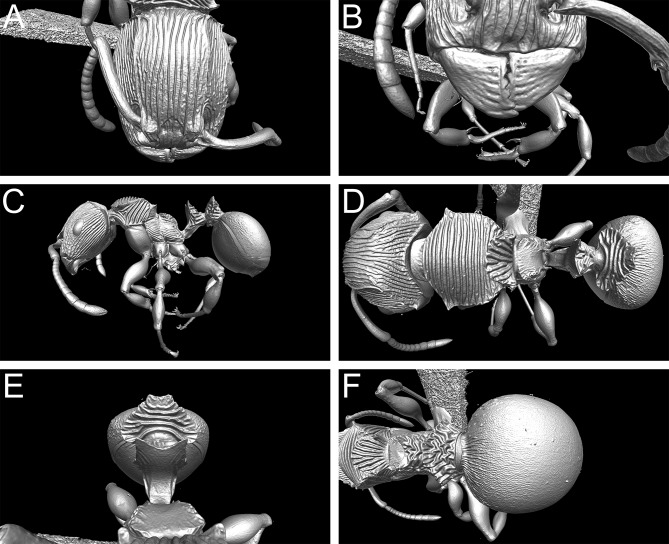
3D surface model of *Terataner balrog* sp. n. holotype worker (CASENT0472559). Shaded surface display volume renderings extracted from 3D-scans. Head in full-face view (A), Anterior head with mandibles (B), body in profile (C), body in dorsal view (D), petiole and postpetiole in anterodorsal view (E), and postpetiole and gaster in dorsal view (F).

**Fig 6:**
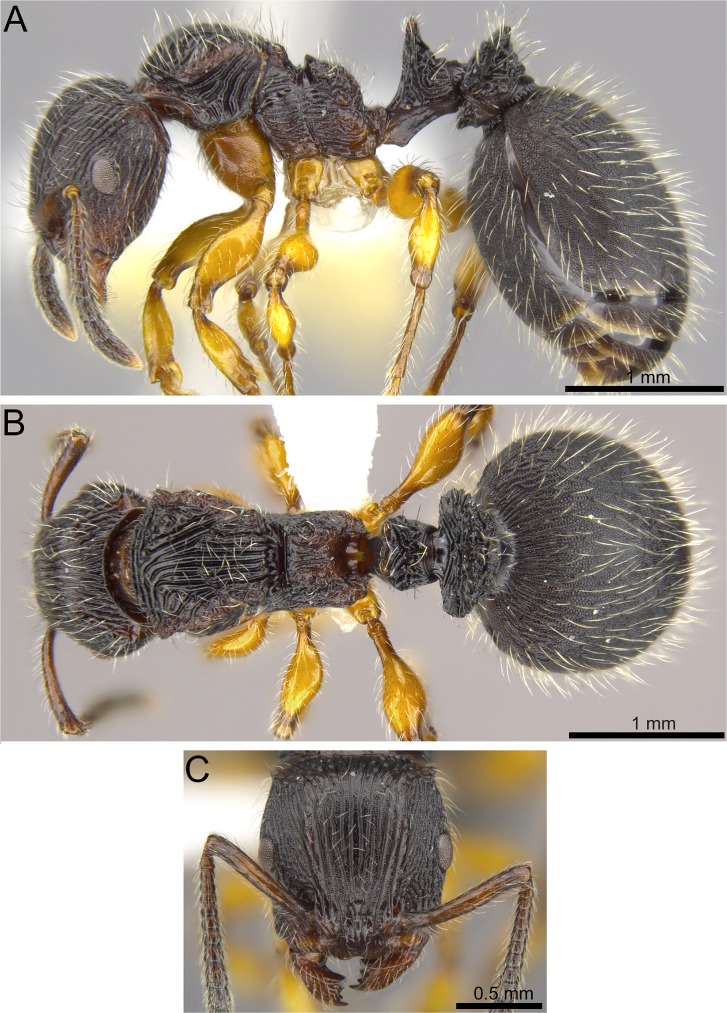
*Terataner balrog* sp. n. paratype ergatoid queen (CASENT0426614). Body in profile (A), body in dorsal view (B), head in full-face view (C).

**Fig 7:**
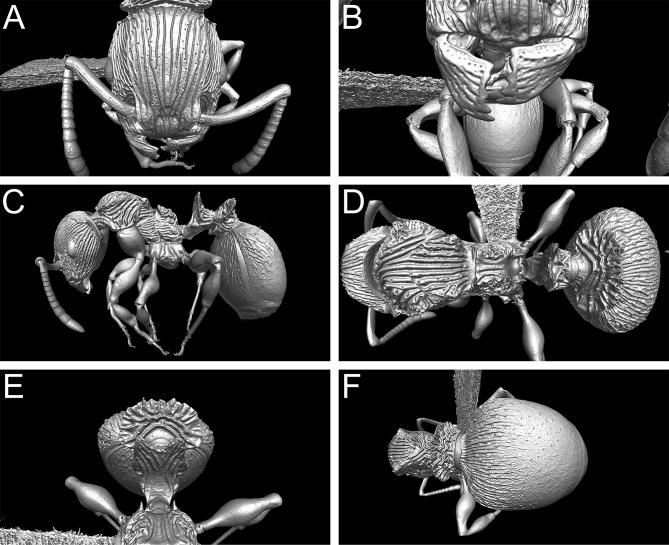
3D surface model of *Terataner balrog* sp. n. paratype ergatoid queen (CASENT0426614). Shaded surface display volume renderings extracted from 3D-scans. Head in full-face view (A), Anterior head with mandibles (B), body in profile (C), body in dorsal view (D), petiole and postpetiole in anterodorsal view (E), and postpetiole and gaster in dorsal view (F).

#### Type material

**Holotype**, pinned worker, MADAGASCAR, Mahajanga, Parc National Tsingy de Bemaraha, 2.5 km 62° ENE Bekopaka, Ankidrodroa River, -19.13222°, 44.81467°, 100 m, tropical dry forest on tsingy, sifted litter (leaf mould, rotten wood), collection code BLF04340, 11.XI.2001 (*B*.*L*. *Fisher et al*.) (CASC: CASENT0472559).

**Paratypes**, 25 pinned workers and one ergatoid queen: two pinned workers with same data as holotype (CASC: CASENT0472560; CASENT0472561); six workers and one ergatoid queen with same data as holotype but collected from rotten tree stump and collection code BLF04373 (CASC: CASENT0426613; CASENT0426614; CASENT0426617; MCZC: CASENT0426612); and 17 workers from Mahajanga, Parc National Tsingy de Bemaraha, 3.4 km 93° E Bekopaka, Tombeau Vazimba, -19.14194°, 44.828°, 50 m, tropical dry forest, collected ex rotten log and dead twig, and as ground foragers, collection codes BLF04238, BLF04283, and BLF04296, 6.XI.2001 (*B*.*L*. *Fisher et al*.) (BMNH: CASENT0426920; CASC: CASENT0426921; CASENT0426922; CASENT0426923; CASENT0426983; CASENT0426984; CASENT0426985; CASENT0426986; CASENT0426987; CASENT0426988; CASENT0427001; CASENT0746134; CASENT0746136; HLMD: CASENT0426926; MCZC: CASENT0426924).

**Cybertypes**, volumetric raw data, 3D PDFs, and 3D rotation videos of the physical holotype (CASC: CASENT0472559) and the paratype ergatoid queen (CASC: CASENT0426614) in addition to montage photos illustrating head in full-face view, profile and dorsal views of the body of both specimens. The data is deposited in Dryad (http://dx.doi.org/10.5061/dryad.sk6s0) and can be freely accessed as virtual representations of the types.

#### Non-type material

MADAGASCAR: Mahajanga, Parc National Tsingy de Bemaraha, 3.4 km 93° E Bekopaka, Tombeau Vazimba, -19.14194°, 44.828°, 50 m, tropical dry forest, ex rotten log and dead twig, 6.XI.2001 (*B*.*L*. *Fisher et al*.); Mahajanga, Parc National Tsingy de Bemaraha, 2.5 km 62° ENE Bekopaka, Ankidrodroa River, -19.13222°, 44.81467°, 100 m, tropical dry forest on tsingy, sifted litter, 11.XI.2001 (*B*.*L*. *Fisher et al*.); Toliara, 48km ENE Morondava, -20.06667°, 44.65°, 30m, tropical dry forest, ground forager(s), 8.XII.1990 (*P*.*S*. *Ward*); Toliara, 50 kms N Morondava, -20.06667°, 44.58333°, in primary dry forest, 13.XII.1991 (*A*. *Pauly*); Toliara, 48 km ENE Morondava, Kirindy Forest, -20.07861°, 44.67556°, 100m, tropical dry forest, root nest on ground, 19.XII.1993 (*G*.*D*. *Alpert*); Toliara, 48km ENE Morondava, Kirindy Forest, -20.074583°, 44.67611°, 100m, tropical dry forest, twig nest 1m off ground, 20.XII.1993 (*G*.*D*. *Alpert*); Toliara, 48 km ENE Morondava, Kirindy, -20.06667°, 44.65°, 30m, tropical dry forest, on low vegetation, 10.XII.1994 (*B*.*L*. *Fisher*); Toliara, Forêt de Kirindy, 15.5 km 64° ENE Marofandilia, -20.045°, 44.66222°, 100m, tropical dry forest, sifted litter, 28.XI.2001 (*B*.*L*. *Fisher et al*.); Toliara, Forêt de Kirindy, 15.5 km 64° ENE Marofandilia, -20.06855°, 44.659565°, 30m, tropical dry forest, Malaise trap, 17.XII.2008 (*B*.*L*. *Fisher*).

#### Diagnosis

*Terataner balrog* can be diagnosed by the following combination of characters: in full-face view head subrectangular and barely longer than wide (CI 93–97); petiole and postpetiole without long, dorsal spines; postpetiole in profile relatively thin and anteroposteriorly compressed, with high and acute triangular dorsum; postpetiole in dorsal view extremely wide, shield-like with sharp transverse crest, apex of dorsum bilobate; dorsum of pronotum conspicuously transversely sulcate/porcate; petiole and postpetiole conspicuously sculptured.

#### Worker measurements (N = 15)

HL 1.18–1.43 (1.28); HW 1.11–1.33 (1.20); FLD 0.50–0.60 (0.55); SL 0.94–1.03 (0.99); EL 0.28–0.33 (0.30); OMD 0.38–0.50 (0.45); WL 1.46–1.84 (1.64); PH 0.52–0.64 (0.59); PW 0.96–1.16 (1.06); PML 0.62–0.76 (0.69); PDH 0.54–0.64 (0.59); MFL 0.94–1.24 (1.10); MFW 0.30–0.38 (0.35); PTH 0.51–0.68 (0.60); PTL 0.30–0.40 (0.35); PTW 0.39–0.53 (0.45); PPH 0.56–0.71 (0.63); PPL 0.28–0.36 (0.32); PPW 0.64–0.85 (0.72); CI 93–97 (94); FLI 45–48 (46); OI 24–29 (25); OMI 64–74 (68); SI 77–89 (82); DMI 63–67 (64); LMI 34–38 (36); PMI 145–163 (154); PPDI 96–104 (100); MFLI 84–99 (91); HFI 304–338 (316); LPeI 54–66 (59); DPeI 113–140 (126); LPpI 44–56 (51); DPpI 205–234 (224).

#### Worker description

HEAD. Masticatory margin of mandible with five or six teeth, usually with large, acute apical tooth followed by a smaller, acute subapical tooth, remaining three to four teeth conspicuously smaller and less acute, very often worn down to small, rounded remnants. Palp formula 5,3. Head in full-face view subrectangular and weakly longer than broad (CI 93–97), sides of head convex and weakly broader behind eye level; posterior head margin weakly rounded; anterior clypeal margin medially with distinct, broad but shallow impression; frontal carinae very well developed, from level of posterior frontal lobes to shortly before posterior head margin subparallel and moderately raised, posteriorly sharply divergent leading to posterolateral corners and stronger raised, crest-like, separating side of head from posterodorsal head; posterior corners with very sharp, strongly raised carinae shortly before occipital margin, dorsalmost point of carinae shaped into short, conspicuous, acute teeth/horns, teeth/horns visible in full-face view. Antennal scrobes strongly reduced to almost absent; antennal scapes moderately long, not reaching posterior head margin (SI 77–89). Eyes of moderate size (OI 24–29), malar area around 1.4 to 1.6 times longer than maximum eye length (OMI 64–74).

MESOSOMA. In lateral view mesosomal outline moderately low (LMI 34–38) with conspicuously convex promesonotum separated from convex propodeum by noticeable but shallow metanotal groove, pronotum approximately of same height as propodeum (PPDI 96–104); promesonotal suture present laterally and completely absent dorsally (pronotum and mesonotum dorsally clearly separated by different surface sculpture–see below); metanotal groove conspicuously developed, shallowly and broadly impressed, in profile anterodorsal propodeal margin weakly overhanging metanotal groove. Pronotum strongly marginate from lateral to dorsal mesosoma; anterodorsally denticulate with pair of large, triangular, and blunt horns laterally; mesonotum only weakly marginate and armed with pair of short, thick and blunt acute teeth laterally; in dorsal view promesonotum anteriorly around 1.4 to 1.6 times wider than long (PMI 145–163). Propodeum strongly marginate from sides to dorsum and sides to propodeal declivity, propodeum unarmed, posterodorsal corners angulate; propodeal lobes well developed, broadly triangular, and blunt. All femorae well swollen medially; length of metafemur around 3.0 to 3.4 times longer than maximum width (HFI 304–338).

WAIST SEGMENTS & GASTER. Petiolar node conspicuously triangular cuneiform and relatively high, with two short, thick, and blunt dorsal teeth; in profile petiolar node approximately 1.5 to 1.9 times higher than long (LPeI 54–66); in dorsal view node approximately ua1.1 to 1.4 times wider than long (DPeI 113–140). Postpetiole in profile relatively thin and anterodorsally compressed, with high and acute triangular dorsum, around 1.8 to 2.3 times higher than long (LPpI 44–56), in anterodorsal view extremely wide, shield-like with sharp transverse crest, apex of dorsum bilobate, postpetiole in dorsal view between 2.0 to 2.3 times wider than long (DPpI 205–234). Gaster not extremely enlarged.

SCULPTURE. Mandibles unsculptured and smooth to weakly rugose/rugulose; cephalic dorsum almost completely longitudinally rugose with more or less regularly arranged rugae, rugae slightly weaker laterally in frontal scrobe area adjacent to frontal carinae; malar area reticulate-rugose; microsculpture on head mostly weakly punctate, especially pronounced between frontal carinae and in scrobal area, absent on clypeus and mandibles. Mesosoma very conspicuously rugose to porcate; dorsum of pronotum conspicuously transversely sulcate/porcate; lateropronotum with very thick, diagonally arranged, parallel sulcate carinae (usually six to seven); dorsal mesonotum longitudinally, partly radiating, sulcate/porcate; lateral mesonotum and propodeum longitudinally rugose; dorsum of propodeum and propodeal declivity unsculptured, smooth and shiny; microsculpture on mesosoma weakly to moderately reticulate-punctate; legs unsculptured, smooth, and shining. Peduncle unsculptured, smooth and shiny; petiolar node anteriorly only weakly sculptured with faint rugae; node laterally and dorsally conspicuously sulcate/porcate, sulci usually arranged longitudinally, sometimes irregularly so; anterior face of postpetiole transversely sulcate, posterior face with longitudinally to irregularly sulcate; microsculpture on both waist segments weakly to moderately reticulate-punctate. Whole gaster with very noticeable and dense reticulate-punctate to punctate microsculpture, base of first gastral tergite longitudinally rugose/rugulose.

PILOSITY & PUBESCENCE. Whole body covered with abundant, short to moderately long, fine, suberect to erect pilosity; pilosity on head noticeably shorter; mandibles with appressed to decumbent hairs; antennal scapes with abundant, shorter, decumbent to suberect hairs on all sides, and often also with row of widely spaced, much longer, erect hairs on anterior margin. Pubescence strongly reduced to absent.

COLORATION. Head, mesosoma, waist segments, and gaster very dark brown to black, antennae and mandibles dark brown, legs yellowish orange to very light brown.

#### Ergatoid queen measurements (N = 5)

HL 1.11–1.14 (1.12); HW 1.00–1.10 (1.04); FLD 0.51 (0.51); SL 0.75–0.82 (0.78); EL 0.22–0.26 (0.24); OMD 0.36–0.40 (0.38); WL 1.40–1.56 (1.49); PH 0.54–0.62 (0.59); PW 0.91–0.96 (0.94); PML 0.68–0.73 (0.71); PDH 0.57–0.62 (0.60); MFL 0.89–0.94 (0.94); MFW 0.25–0.28 (0.27); PTH 0.60–0.64 (0.62); PTL 0.31–0.35 (0.33); PTW 0.45–0.53 (0.50); PPH 0.61–0.70 (0.65); PPL 0.25–0.33 (0.30); PPW 0.81–0.85 (0.82); CI 90–96 (93); FLI 46–51 (49); OI 21–24 (22); OMI 59–69 (63); SI 72–76 (74); DMI 61–65 (63); LMI 39–40 (39); PMI 130–134 (132); PPDI 94–103 (97); MFLI 86–89 (87); MFI 321–357 (341); LPeI 50–56 (53); DPeI 129–158 (144); LPpI 36–52 (46); DPpI 249–340 (282).

#### Ergatoid queen description.

HEAD. Masticatory margin of mandible with five teeth, usually with large, acute apical tooth followed by a smaller, acute subapical tooth, remaining three teeth conspicuously smaller and less acute, very often worn down to small, rounded remnants. Palp formula 5,3. Head in full-face view subrectangular and weakly longer than broad (CI 90–96), sides of head convex and weakly broader behind eye level; posterior head margin weakly rounded; anterior clypeal margin medially with distinct, broad but shallow impression; frontal carinae very well developed, from level of posterior frontal lobes to shortly before posterior head margin subparallel and moderately raised, posteriorly sharply divergent leading to posterolateral corners and stronger raised, crest-like, separating side of head from posterodorsal head; posterior corners with very sharp, strongly raised carinae shortly before occipital margin, dorsalmost point of carinae shaped into short, conspicuous, acute teeth/horns, teeth/horns visible in full-face view. Antennal scrobes strongly reduced to almost absent; antennal scapes short to moderately long, not reaching posterior head margin (SI 72–76). Eyes of moderate size (OI 21–24), malar distance around 1.4 to 1.7 times longer than maximum eye length (OMI 59–69).

MESOSOMA. In lateral view mesosomal outline moderately low (LMI 39–40) with conspicuously convex promesonotum separated from convex propodeum by noticeable but shallow metanotal groove, pronotum approximately of same height as propodeum (PPDI 94–103); promesonotal suture present laterally and completely absent dorsally (pronotum and mesonotum dorsally partly separated by different surface sculpture); metanotal groove conspicuously developed, shallowly and broadly impressed, in profile anterodorsal propodeal margin weakly overhanging metanotal groove. Pronotum strongly marginate from lateral to dorsal mesosoma; anterodorsally denticulate with pair of moderately large, triangular, and blunt horns laterally; mesonotum only weakly marginate and armed with pair of short, thick and blunt acute teeth laterally; in dorsal view promesonotum anteriorly around 1.3 times wider than long (PMI 130–134). Propodeum strongly marginate from sides to dorsum and sides to propodeal declivity, propodeum unarmed, posterodorsal corners angulate; propodeal lobes well developed, broadly triangular, and blunt. All femorae well swollen medially; length of metafemur around 3.2 to 3.6 times longer than maximum width (HFI 321–357).

WAIST SEGMENTS & GASTER. Petiolar node conspicuously triangular cuneiform and relatively high, with two short, thick, and blunt dorsal teeth; in profile petiolar node around 1.8 to 2.0 times higher than long (LPeI 50–56); in dorsal view node around 1.3 to 1.6 times wider than long (DPeI 129–158). Postpetiole in profile relatively thin and anterodorsally compressed, with high and acute triangular dorsum, around 1.9 to 2.8 times higher than long (LPpI 36–52), in anterodorsal view extremely wide, shield-like with sharp transverse crest, apex of crest variably shaped, ranging from roughly bilobate or thickly serrated to smooth and rounded, postpetiole in dorsal view between 2.5 to 3.4 times wider than long (DPpI 249–340). Gaster extremely enlarged.

SCULPTURE. Mandibles unsculptured and smooth; cephalic dorsum almost completely conspicuously longitudinally rugose with more or less regularly arranged rugae, rugae slightly weaker laterally in frontal scrobe area adjacent to frontal carinae; malar area reticulate-rugose; microsculpture on head conspicuously punctate, especially pronounced between frontal carinae and in scrobal area, absent on clypeus and mandibles. Mesosoma very conspicuously rugose to porcate; dorsum of pronotum and mesonotum variably sculptured, ranging from mostly transversely sulcate/porcate pronotal dorsum with irregular elements merging to mostly longitudinally sulcate/porcate with irregular elements to completely irregularly sulcate/porcate on both; lateropronotum with very thick, diagonally arranged, parallel sulcate carinae (usually four); lateral mesonotum and propodeum longitudinally rugose; dorsum of propodeum weakly rugose; propodeal declivity unsculptured, smooth and shiny; microsculpture on mesosoma weakly to moderately reticulate-punctate; legs unsculptured, smooth, and shining. Peduncle unsculptured, smooth and shiny; petiolar node anteriorly only weakly sculptured with faint rugae; node laterally and dorsally conspicuously sulcate/porcate, sulci usually arranged longitudinally, sometimes irregularly so; anterior face of postpetiole transversely sulcate, posterior face with longitudinally to irregularly sulcate; microsculpture on both waist segments weakly to moderately reticulate-punctate. Whole gaster with very noticeable and dense reticulate-punctate to punctate microsculpture, basal third of first gastral tergite longitudinally rugose/rugulose.

PILOSITY & PUBESCENCE. Whole body covered with abundant, moderately long, fine, suberect to erect pilosity; pilosity on head shorter; mandibles with subdecumbent to suberect hairs; antennal scapes with abundant, shorter, decumbent to suberect hairs on all sides, and often also with row of widely spaced, much longer, erect hairs on anterior margin. Pubescence strongly reduced to absent.

COLORATION. Similar to worker caste but head and mesosoma with patches of much lighter brown, especially dorsolateral margins of pronotum.

#### Intercaste variation

Among the material of the new species available to us, we identified four ergatoid queens that are morphologically close to the worker caste. Nevertheless, despite this superficial similarity, the ergatoids are separable from the workers. Within the examined colonies the ergatoid queens turned out to be the smallest adult females that were conspicuously much hairier and of lighter colour than the workers. In addition, among other characters, the ergatoid reproductives possess shorter antennal scapes, smaller eyes, smaller anterolateral horns/teeth on the pronotal dorsum, a less broad promesonotum, thicker hind femorae, a broader petiolar node, thicker dorsal petiolar teeth, a broader postpetiole, a significantly much larger gaster, and different sculpture (for a detailed list of intercaste differences see Table 3). It remains unclear if the differences observed in *T*. *balrog* are typical for the whole genus. On the basis of a first examination of ergatoids from more than a dozen species, it appears as if some worker-ergatoid differences will be observable in all or most species, such as the smaller body size and eyes, shorter antennal scapes, thicker dorsal petiolar teeth/spines (if present in the species), wider and generally more massive postpetiole, as well as lighter body colour and irregularly arranged surface sculpture. Nevertheless, general trends and patterns of ergatoid queen versus worker morphologies will be assessed in detail in a future revision of the whole genus.

**Table 3:** Intercaste variation. Morphometric and morphological characters showing key differences between the worker and the ergatoid reproductive castes of *T*. *balrog* **sp. n.** Morphometric data is presented in parentheses as minimum and maximum values.

Worker caste	Ergatoid queen caste
usually larger body size (HW 1.13–1.33; WL 1.46–1.84)	usually smaller body size (HW 1.00–1.10; WL 1.40–1.56)
longer antennal scapes (SI 77–89)	slightly shorter antennal scapes (SI 72–76)
slightly larger eyes (OI 24–29)	slightly smaller eyes (OI 21–24)
promesonotal dorsum anteriorly broader (PMI 145–163)	promesonotal dorsum anteriorly thinner (PMI 130–134)
slightly thinner metafemorae (HFI 304–338)	slightly thicker metafemorae (HFI 321–357)
in dorsal view petiolar node less broad (DPeI 113–140)	in dorsal view petiolar node broader (DPeI 129–158)
dorsal petiolar teeth thinner	dorsal petiolar teeth thicker
in anterodorsal view postpetiole more triangular and less massive	in anterodorsal view postpetiole arc-like and much more massively shaped
postpetiole in dorsal view less broad (DPpI 205–234)	postpetiole in dorsal view much broader (DPpI 249–340)
gaster not greatly enlarged	gaster greatly enlarged
pronotal dorsum always transversely sculptured and mesonotal dorsum longitudinally sculptured	promesonotal dorsum variably sculptured, usually highly irregularly sculptured
lateropronotum with six to seven thick, diagonally arranged, parallel sulci	lateropronotum usually with four very thick, diagonally arranged, parallel sulci
less hairy	much hairier
head and mesosoma of darker colour	head and mesosoma of lighter colour

#### Etymology

The new species is named after the fictional evil creature from J. R. R. Tolkien’s “Lord of the Rings” trilogy in reference to the “dark” predatory lifestyle common within the genus *Terataner*, as well as its strongly armoured and horned gestalt. The species epithet is a noun in apposition and thus invariant.

#### Distribution and biology.

*Terataner balrog* is known from Tsingy de Bemaraha and Kirindy in arid, western Madagascar where it was sampled from tropical dry forest habitats. It seems to live in twigs and branches on low vegetation above ground, as well as in tree stumps on the ground and in roots in the ground. Foragers can be found in the lower vegetation and in smaller numbers also on the ground.

#### Diagnostic discussion

The new species is a highly distinguishable member of the Malagasy fauna and cannot be confused with any congener. Of special importance are the strongly transverse porcate sculpture on the pronotal dorsum and the shape of petiole and postpetiole. The sculpture on the pronotal dorsum appears to be unique at present since we did not observe it in another congeneric species. Not considering the sculpture, one can also easily separate *T*. *balrog* from all other *Terataner* on the basis of the postpetiolar shape.

#### Intraspecific variation

The worker caste of *T*. *balrog* is remarkably uniform with no significant, observable intraspecific variation. There is some variation in body size (WL 1.46–1.84), which is well within the normal range of many myrmicine ants. The ergatoid queens display more variation, as can be seen from the above description. Especially the shape of the postpetiolar dorsum and the sculpture on the dorsal promesonotum are unique in every single specimen studied and vary to a great extent between them.

### *Terataner nymeria* Hita Garcia sp. n.

**Fig 8:**
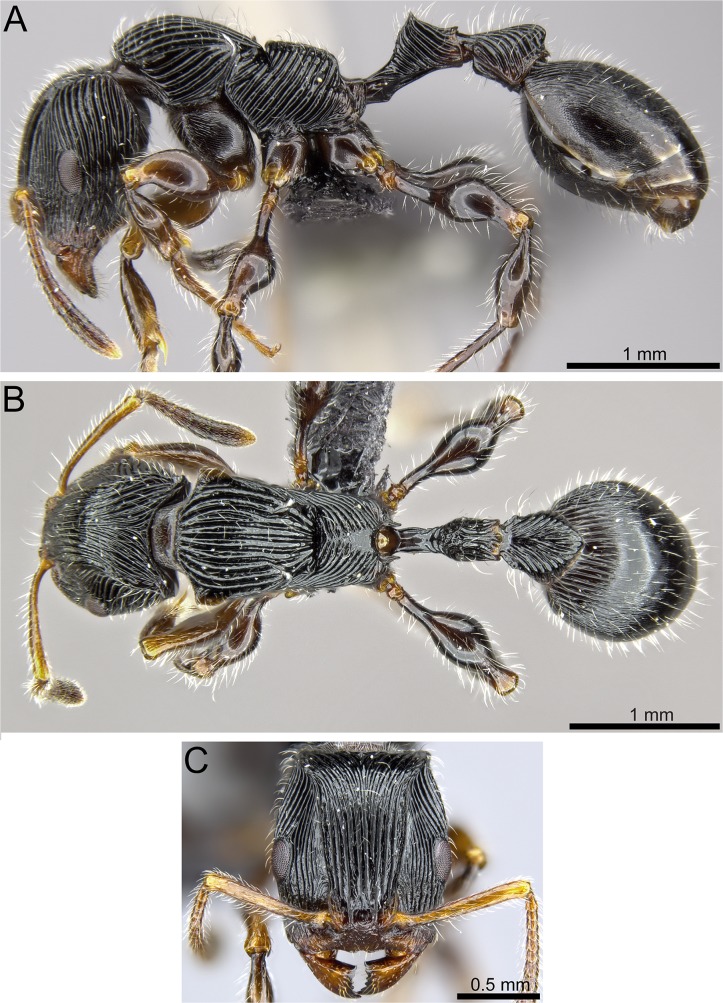
*Terataner nymeria* sp. n. holotype worker (CASENT0053630). Body in profile (A), body in dorsal view (B), head in full-face view (C).

**Fig 9:**
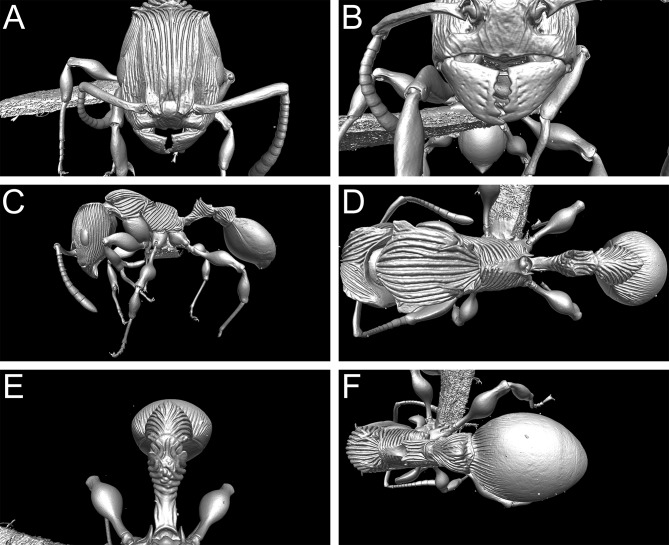
3D surface model of *Terataner nymeria* sp. n. holotype worker (CASENT0053630). Shaded surface display volume renderings extracted from 3D-scans. Head in full-face view (A), Anterior head with mandibles (B), body in profile (C), body in dorsal view (D), petiole and postpetiole in anterodorsal view (E), and postpetiole and gaster in dorsal view (F).

**Fig 10:**
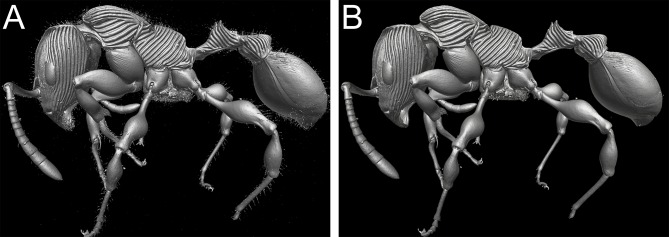
Virtual recovery of pilosity of *T*. *nymeria* sp. n. Shaded surface display volume renderings extracted from 3D-scans with different settings of colour range: showing pilosity with background noise (A), without pilosity after reduction of background noise (B).

urn:lsid:zoobank.org:act:4049D328-38A9-4CC2-816B-472155CB2D40

(Figs 1, 3C, 3F–3G and 8–10, S3 Fig, S3 Video)

#### Type material

**Holotype**, pinned worker, MADAGASCAR, Antsiranana, Reserve Analamerana, 16.7 km 123° Anivorano-Nord, -12.80467°, 49.37383°, 225 m, tropical dry forest, on low vegetation, collection code BLF11298, 3.XII.2004 (*B*.*L*. *Fisher*) (CASC: CASENT0053630).

**Paratypes**, eleven pinned workers: eight workers with same data as holotype except collected ex dead branch above ground, bamboo and collection code BLF11305 (BMNH: CASENT0790128; CASC: CASENT0107418; CASENT0746053; CASENT0746054; CASENT0746055; CASENT0790129; HLMD: CASENT0790127; MCZC: CASENT0790130); and one worker with same data as holotype except collected by beating low vegetation and collection code BLF11306 (CASENT0107444).

**Cybertype**, volumetric raw data, 3D PDF, and 3D rotation video of the physical holotype (CASC: CASENT0053630) plus montage images illustrating head in full-face view, profile and dorsal views of the body. The data of the holotype is deposited in Dryad (http://dx.doi.org/10.5061/dryad.sk6s0) and can be freely accessed as virtual representation of the physical holotype.

#### Diagnosis

The following character combination distinguishes *T*. *nymeria* from the remainder of the genus: in full-face view head comparatively thin (CI 87–91) with weakly convex sides, broadest at eye level, conspicuously narrowing towards posterior head margin before significantly broadening again into well-developed angulate posterodorsal corners; petiole and postpetiole without extremely long, dorsal spines; postpetiole in profile strongly rounded cuneiform, anterior face relatively long and straight leading to rounded dorsum, dorsal apex slightly pointing backwards; postpetiole in anterodorsal view arch-like leading towards a rounded apex; dorsum of promesonotum longitudinally porcate; petiole and postpetiole conspicuously sculptured.

#### Worker measurements (N = 12)

HL 1.08–1.21 (1.13); HW 0.98–1.08 (1.01); FLD 0.36–0.45 (0.41); SL 0.75–0.86 (0.81); EL 0.26–0.30 (0.28); OMD 0.35–0.38 (0.37); WL 1.56–1.76 (1.62); PH 0.52–0.62 (0.55); PW 0.81–0.97 (0.97); PML 0.75–0.86 (0.80); PDH 0.54–0.61 (0.57); MFL 0.98–1.10 (1.02); MFW 0.31–0.36 (0.32); PTH 0.40–0.49 (0.43); PTL 0.47–0.51 (0.49); PTW 0.27–0.33 (0.29); PPH 0.50–0.58 (0.52); PPL 0.47–0.56 (0.50); PPW 0.48–0.58 (0.52); CI 87–91 (90); FLI 36–43 (40); OI 26–29 (28); OMI 70–83 (77); SI 75–82 (80); DMI 52–56 (54); LMI 33–35 (34); PMI 106–113 (110); PPDI 93–101 (97); MFLI 99–104 (101); MFI 306–332 (316); LPeI 105–123 (115); DPeI 55–64 (58); LPpI 91–99 (95); DPpI 99–111 (104).

#### Worker description

HEAD. Masticatory margin of mandible with five or six teeth, usually with large, acute apical tooth followed by a smaller, acute subapical tooth, remaining three to four teeth conspicuously smaller and less acute, very often worn down to small, rounded remnants. Palp formula 5,3. Head in full-face view comparatively thin, around 1.1 times longer than wide (CI 87–91), sides of head weakly convex, broadest at eye level, conspicuously narrowing towards posterior head margin before significantly broadening again into well-developed angulate posterodorsal corners; in full-face view posterior head margin straight to very weakly convex; anterior clypeal margin medially with distinct, broad but small impression; frontal carinae very well developed, from level of posterior frontal lobes to shortly before posterior head margin subparallel and moderately raised, posteriorly initially sharply divergent and much more raised, crest-like, merging with strong costate sculpture and separating side of head from posterodorsal head. Antennal scrobes present but very shallow and narrow; antennal scapes moderately long, not reaching posterior head margin (SI 75–82). Eyes of moderate size (OI 26–29), malar distance around 1.2 to 1.4 times longer than maximum eye length (OMI 70–83).

MESOSOMA. In lateral view mesosoma relatively low and elongate (LMI 33–35), promesonotum moderately convex, propodeal dorsum weakly convex, propodeum usually weakly higher than propodeum (PPDI 93–101) and both separated by metanotal groove; promesonotal suture present laterally and completely absent dorsally, metanotal groove well developed, moderately deep but relatively narrow, in profile anterodorsal propodeal margin weakly overhanging metanotal groove. Pronotum strongly marginate from lateral to dorsal mesosoma; anterodorsal corners shaped into sharply rectangular, thin, and transparent lamellae, lamellae becoming weaker and less transparent posteriorly and ending at promesonotal suture; mesonotum not marginate and armed with pair of short, thin, and acute teeth laterally; in dorsal view promesonotum around 1.1 times wider than long (PMI 106–113). Propodeum moderately marginate from sides to dorsum and sides to propodeal declivity; propodeum unarmed, posterodorsal corners angulate; propodeal lobes very well developed, broadly triangular, and blunt; in dorsal view anterodorsal margin of propodeum straight. All femorae strongly swollen medially; length of metafemur around 3.1 to 3.3 times longer than maximum width (HFI 306–332).

WAIST SEGMENTS & GASTER. In profile petiolar node relatively low and conspicuously triangular cuneiform; in anterodorsal view node with two short but distinct, blunt dorsal spines; in dorsal view approximately barrel-shaped, sides mostly straight and converging anteriorly towards peduncle; in profile petiolar node around 1.1 to 1.2 times longer than high (LPeI 105–123); in dorsal view node between 1.5 to 1.8 times longer than wide (DPeI 55–64). In profile postpetiole strongly rounded cuneiform, around 1.0 to 1.1 times higher than long (LPpI 91–99), anterior face relatively long and straight leading to rounded dorsum, dorsal apex slightly pointing backwards; in anterodorsal view postpetiole arch-like leading towards a rounded apex; in dorsal view around as wide as long to 1.1 times wider than long (DPpI 99–111). Gaster not extremely enlarged.

SCULPTURE. Mandibles unsculptured, smooth, and shiny; clypeus and anterior head margin only weakly sculptured, usually with few weak, longitudinal carinulae; cephalic dorsum between frontal carinae conspicuously longitudinally costate, thickness and height of costae increasing from anterior margin near clypeus to posterior head; antennal scrobe mostly unsculptured, smooth and shiny; lateral head longitudinally costate, weaker developed anteriorly and between eyes and frontal carinae, becoming much stronger posteriorly. Most of mesosoma very conspicuously porcate; lateropronotum and dorsal promesonotum longitudinally porcate; lateral propodeum diagonally porcate, dorsum anteriorly close to metanotal groove transversely porcate, median area of dorsum and propodeal declivity unsculptured, smooth and shiny; procoxae mostly longitudinally rugulose, remainder of legs unsculptured, smooth and shiny. Peduncle unsculptured, smooth and shiny; both waist segments strongly costate/porcate: sides of petiolar node diagonally costate, most costae crossing anterior side of node from one side to another; dorsum of node longitudinally costate/porcate; postpetiole longitudinally costate. Base of first gastral tergite costulate, remainder of tergite with conspicuous reticulate-punctate microsculpture. Apart from first gastral tergite body with very weak to absent microsculpture.

PILOSITY & PUBESCENCE. Whole body covered with abundant, short to moderately long, fine, suberect to erect pilosity; pilosity on gaster noticeably shorter; mandibles with appressed to decumbent hairs; antennal scapes with abundant, shorter, decumbent to suberect hairs on all sides plus row of widely spaced, much longer, erect hairs on anterior margin. Pubescence strongly reduced to absent.

COLORATION. Most of body very dark brown to black.

#### Etymology

The new species is named after the fictional, female direwolf from Georg R. R. Martin’s epic fantasy series “A Song of Ice and Fire” referring to the ferocious, predatory nature of the new species. The species epithet is a noun in apposition and thus invariant.

#### Distribution and biology

*Terataner nymeria* is only known from its type locality, the Analamerana Special Reserve, which is located in the northern tip of Madagascar. The available material was collected in a dry tropical forest habitat at an altitude of 225 m. The new species seems to live on vegetation and nest in dead branches. The strongly elongate and slender body might be an adaptation to the life in sticks and branches resembling other arboreal stick-inhabiting ants, such as *Tetraponera* Smith.

#### Diagnostic discussion

This new species is distinguishable from all other *Terataner* on the basis of the slender and elongate gestalt. There is no other *Terataner* that comes close, especially when considering the relatively thin and elliptic head shape in full-face view. Some undescribed species from Madagascar have thinner heads compared to most other congeners, but they never have the strongly narrowed posterior head as seen in *T*. *nymeria*. The same is true for the strongly elongate waist segments, which are not found in any other *Terataner*. The phylogenetic affinities of *T*. *nymeria* are unknown at present, but on the basis of its peculiar morphology it seems that it is not closely related to any other currently described *Terataner* species.

#### Intraspecific variation

Considering that *T*. *nymeria* is only known from three collections from the same locality on the same day, it is not surprising that intraspecific variation is neglectable.

### Microtomography and cybertaxonomy

#### Exploration of μCT data for ant taxonomy

Our experience utilizing μCT for a taxonomic analysis of *Terataner* species highlights both opportunities and challenges for the new technology going forward. As was the case for land planarians, millipedes, and centipedes [13, 40, 42], the volumetric μCT data used in this study was sufficient and adequate for the characterization and taxonomic discrimination of these two new ant species. Most external morphological information needed for species-level taxonomy, such as body size, dimensions, general shape, and overall surface sculpture are as accurate in the μCT scans (Figs 5, 7 and 9) as in the physical specimens (Figs 4, 6 and 8) allowing a straightforward identification of *T*. *balrog* and *T*. *nymeria* from their congeners. In order to demonstrate that μCT scans are reliable 3D reconstructions of the physical material, we measured the physical specimens with an optical microscope and the virtual 3D models with basic software measuring tools. Our results show that there are no significant differences between the physical and the virtual specimens (Table 4). The error rates for repeated measures are on average 0.004 for all three specimens, which is in the range of normal measurement error. We used commonly available software such as Adobe Acrobat Pro, to make the measurements on the 3D PDF, although it was at times more cumbersome than traditional measurements under the microscope. On the other hand, the 3D model has the advantage that other researchers can replicate measurements and make new measurements without receiving the physical specimen.

**Table 4:** Morphometric comparison of physical versus virtual specimens. Comparison of raw data of all used measurements for the three scanned specimens with minimum, maximum, and average values. Error rate is used as an indicator of accuracy. Abbreviations are as follows: w = worker, q = ergatoid queen, p = physical specimen, v = virtual specimen.

	T. balrog w / p	T. balrog w / v	Error Rate	T. balrog q / p	T. balrog q / v	Error Rate	T. nymeria w / p	T. nymeria w / v	Error Rate
**HL**	1.429	1.420	0.004	1.143	1.148	0.003	1.188	1.186	0.001
**HW**	1.325	1.320	0.003	1.095	1.086	0.005	1.038	1.041	0.002
**FLD**	0.600	0.590	0.005	0.508	0.527	0.010	0.425	0.416	0.005
**SL**	1.025	1.030	0.003	0.820	0.810	0.005	0.838	0.850	0.006
**EL**	0.330	0.321	0.005	0.260	0.250	0.005	0.300	0.301	0.001
**OMD**	0.500	0.490	0.005	0.400	0.390	0.005	0.375	0.379	0.002
**WL**	1.840	1.835	0.002	1.556	1.550	0.003	1.700	1.700	0.000
**PH**	0.640	0.660	0.010	0.619	0.620	0.000	0.560	0.575	0.007
**PW**	1.163	1.150	0.006	0.950	0.960	0.005	0.920	0.920	0.000
**PML**	0.762	0.760	0.001	0.730	0.730	0.000	0.840	0.836	0.002
**PDH**	0.640	0.650	0.005	0.603	0.610	0.003	0.560	0.580	0.010
**HFL**	1.238	1.210	0.014	0.938	0.930	0.004	1.038	1.050	0.006
**HFW**	0.375	0.370	0.003	0.263	0.270	0.004	0.313	0.317	0.002
**PTH**	0.683	0.680	0.002	0.625	0.623	0.001	0.450	0.441	0.005
**PTL**	0.397	0.390	0.003	0.313	0.320	0.004	0.490	0.481	0.004
**PTW**	0.530	0.530	0.000	0.495	0.480	0.008	0.290	0.284	0.003
**PPH**	0.700	0.700	0.000	0.700	0.700	0.000	0.540	0.528	0.006
**PPL**	0.363	0.360	0.001	0.250	0.260	0.005	0.505	0.500	0.003
**PPW**	0.850	0.840	0.005	0.850	0.840	0.005	0.500	0.492	0.004
**Minimum**			**0.000**			**0.000**			**0.000**
**Maximum**			**0.014**			**0.010**			**0.010**
**Average**			**0.004**			**0.004**			**0.004**

Nevertheless, despite the great morphological accuracy and fidelity, the volumetric μCT data does not provide a 100% representation of the physical specimens and there are some limitations that could be problematic for certain taxonomic cases. The most obvious weakness of the μCT scans is the lack of natural body colour on the specimen surface. At present, it is not possible to detect true colours by any microtomography technique [30], even though this might be achievable in the near future through the use of hybrid true-colour μCT [62]. In addition, the fine punctate surface microsculpture typical of many ants and most *Terataner* is generally well recovered in the scans, but in a few instances of the three scanned specimens it appears slightly reduced, whereas it is clearly present and conspicuous in the physical specimens. The situation for pilosity is problematic as well. In the case of the *T*. *nymeria* scan, the very fine hairs are hard to see in the reconstructed data since they are mostly obscured by background noise (Fig 10A). After reduction of this noise with the visualisation software Amira in order to create a better resolved 3D model, no hairs remain visible (Fig 10B). Concerning both scans of *T*. *balrog*, pilosity is better recovered and can be examined more in detail since hairs are generally thicker in this species compared to *T*. *nymeria*. However, it is imperfect and is less discernible with a higher degree of background noise on most of the head, legs and some parts of the gaster. The poor resolution of fine hairs in insects is not new and was already reported for bees by Greco et al. [63]. Another disadvantage encountered in the scans is that most ommatidia are not well discernible. While the ommatidia of the *T*. *balrog* queen are very distinctive in the scans (Fig 7A and 7C), they are less defined in the *T*. *nymeria* holotype (Fig 9A and 9C), and almost unperceivable in the holotype of *T*. *balrog* (Fig 5A and 5C). This lack of resolution for compound eyes is in accordance with results for land planarians [42] and bees [63].

With the exception of body colour, all of the above limitations are related to limitations in isotropic voxel resolution, and more specifically the trade-off between resolution and field-of-view. This was a problem in previous studies using μCT scanning that encountered similar difficulties with the visualisation of smaller external and/or internal structures [13, 30, 42, 43, 63, 64]. With most of the currently available μCT systems isotropic voxel resolution is limited to a range of around 1–100 μm [30], and most of the aforementioned studies employing μCT data for taxonomy used voxel sizes of approximately 2–10 μm with this study being well within that range (ca. 5 μm). Although the available maximum resolution is sufficient for the visualisation of most of the external morphology and/or internal anatomy, it is not sufficient for minute structures [30, 64]. Previous studies using nanoCT on gastropods [65] and synchroton-based μCT on mites [36, 66] achieved highly resolved data rich in details of microscopic structures, otherwise only obtainable through SEM. Our instrument can operate at a comparatively high resolution (approximate maximum of 0.3 um), however higher spatial resolution comes at a cost of total field of view thus it can be a challenge to scan whole ant specimens at full resolution. Future technological advancements may provide solutions for the trade-off between resolution and field of view, and could provide volumetric datasets with higher resolution and increased visibility of smaller external or anatomical structures, and might be able to recover pilosity and microsculpture.

Fortunately, the weaknesses in μCT for external morphology correspond to the strengths of montage photos taken under an optical microscope, which are currently the standard accompaniment to ant species descriptions. The montage photos clearly show pilosity, eye structure, and microsculpture, and so combined with the 3D tomograms give a complete set of characters that are normally used in ant taxonomy in addition to many other aspects of the phenotype that could be of scientific interest.

**Fig 11:**
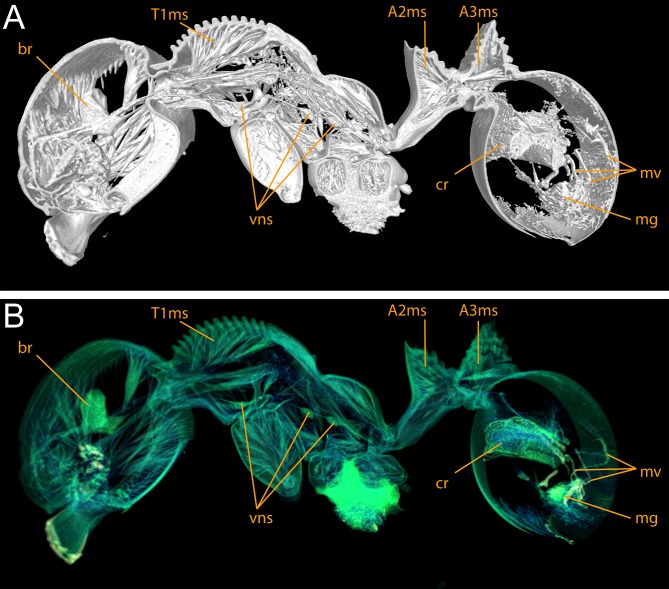
Virtual cross section displaying internal anatomy of *T*. *balrog* sp. n. (CASENT0472559). Shaded surface display volume rendering (A), False colour volume rendered sagittal virtual cross section of (B), Abbreviations: br = brain; vns = ventral nerve system; T1ms = thorax segment 1 muscles; A2ms = abdominal segment 2 muscles; A3ms = abdominal segment 3 muscles; cr = crop; mv = Malpighian vessels; mg = midgut.

While internal structures are not commonly used in ant taxonomy (with some exceptions), it is worth asking how μCT performs at capturing these structures, as they may be of interest to researchers. Internal anatomical structures are partly recognisable in all three scans. The brain and nervous system, muscular structure, and most of the digestive system are visible in the raw data (Fig 11). However, due to the use of dry-mounted museum specimens for this study, most of these structures had undergone shrinking and desiccation prior to scanning and are far from accurate and comparable to living specimens. For some applications, such as biomechanical analysis of muscle and cuticular structures, this may be sufficient. However, other internal structures and/or soft tissues, such as glands, the tracheal system, or the reproductive organs of the queen, were not recoverable or differentiable from surrounding tissues. For a precise, life-like representation of internal structures, recently collected material would have been necessary, either preserved in ethanol, freshly killed, or still alive [63]. Nevertheless, as modern ant taxonomy is almost exclusively based on external morphology of the female castes [50, 67–69], the poor recovery of internal structures in dry specimens is not a hindrance for the use of μCT data as taxonomic data. Future studies focusing on a broader taxon set and using freshly sampled material might provide information about internal characters of high diagnostic value for different taxonomic levels. The sting apparatus, which is present in most ant groups, might have potential as an important diagnostic structure for higher taxonomic levels [70–73], while cuticular and muscular structures in the pronotum of worker ants might have potential for species level diagnostics on the basis of the high importance of thoracic muscles in that body part [74]. Another prospective application not explored in this study is the virtual reconstruction of male genitalia in ants, which normally requires dissection. This process is laborious, time-consuming, and requires a special skillset [75, 76]. The non-destructive application of μCT reconstructions of male genitalia could facilitate the study of male ants and enable a larger number of researchers to use this often neglected caste for systematics.

MicroCT may also be useful for ant taxonomy for other reasons. One issue frequently neglected by ant taxonomy is the potential infection by mermithid nematode parasites that cause a significant alteration of the phenotype. By employing μCT scanning Csősz [77] showed that several species of rarely collected and putatively socially parasitic species were wrongly described as distinct on the basis of a mermithogenic phenotype. In addition, based on some case examples from Europe, Csősz [77] postulated that cases of undetected infections might be more common than anticipated, and not just in socially parasitic species. Thus, the use of μCT data for ant taxonomy can prevent erroneous descriptions of new taxa. Based on the scanning data presented in this study, which does not show any signs of nematode infestation, we can rule out such an infestation for *T*. *balrog* and *T*. *nymeria*.

Another potential benefit of using μCT scanning data for ant taxonomy is the potential to reveal hidden characters. In dry-mounted specimens, and older museum specimens in particular, it is often hard to see certain characters which can be obscured due to outdated mounting methods. This can be due to the excessive use of glue on the paper tip that covers parts of the body, or by having some body parts (usually legs or antennae) hiding other body parts. Hidden characters not visible under normal circumstances usually include mouthparts, mandibular teeth, clypeal structures, parts of the petiole or postpetiole, or leg spurs. By using software to visualise μCT data it is easy to virtually remove parts of the scan and reveal these hidden structures. This can add additional character states for descriptions that would otherwise be unavailable for publication, and uncover characters from many historic type specimens that were previously impossible to use for comparative examinations. This was the case for the palp formula of both new species described here. Due the limited number of specimens, dissection was not an option. Examination of physical specimens provided some approximate count, which we were able to confirm with the scanned data. Furthermore, in this study we used the μCT scans in combination with the physical specimens and montage images in order to study the morphology of both new species, and conclude that the use of a 3D model of a new species can be extremely helpful during the description writing process. It avoids having to handle and expose delicate and rare type material too often, and seeing a scalable, rotatable high-resolution 3D reconstruction on a screen provides different perspectives and a better understanding of morphology compared to the limited depth of field achieved by light microscopes.

#### Cybertypes in invertebrate and ant taxonomy

Perhaps the most important advantage of using μCT data for ant taxonomy is its potential application as virtual avatar or cybertype. The idea behind taxonomic cybertypes is to provide a detailed and as complete as possible virtual reconstruction of the morphology and anatomy of physical type material, which is size-calibrated, virtually dissectible, rotatable, and measurable, thus allowing a high degree of interactive manipulation [12, 13]. Of crucial importance is the non-destructive nature of μCT scanning, which leaves the original, physical type specimens intact and unharmed.

We hasten to add that a cybertype, as defined and used in the literature [12, 13, 40], is more of a concept and not a type designation in a traditional sense; notably it has no nomenclatural value. We do not intend to replace physical types by virtual types in this study nor do we propose to do this in the future. However, at least for normal taxonomic purposes, a cybertype should be nearly functionally equivalent to the physical type, and thus obviate the need to loan or travel to the physical specimen. For normal taxonomic study of morphology, the researcher can work as (or more) effectively with the cybertype as they would with the physical type specimen. As discussed above, the μCT tomogram itself is not sufficient to serve this purpose, due to limitations in capturing pilosity and colour information. However, the complementarity of μCT and montage photos, which excel at capturing colour/pilosity elements but not quantitative information on shape, allows the two types of images to work together effectively. We propose that a minimal cybertype for ant taxonomy should consist of a μCT scan and a set of montage photos from the three standard views (head, profile, dorsal).

Making cybertypes available to the research community provides an opportunity for a more thorough testing of the repeatability and robustness of their published species hypotheses, compared to more traditional taxonomy. It is easier to assess the quality of alpha taxonomy by having as comprehensive data as possible with the currently available technology and it can serve to improve the quality control of taxonomic publications. If reviewers can see the full morphology of the putative new species submitted to peer review, it will be much more difficult to describe new species on the basis of one or two obscured characters not visible or hard to detect in line drawings or two-dimensional light photography. Reviewers also have the possibility to re-measure the virtual specimens and provide a more thorough testing. Furthermore, future taxonomic studies can be accelerated by using virtual cybertypes since, as mentioned above, it is often challenging and time-consuming to organise type material from museum collections. In many cases, it might be sufficient to virtually examine and measure the cybertype to get an idea about heterospecificity of the studied material. Nonetheless, in other cases it might still be necessary to obtain the original type material.

One possibility that deserves discussion is that freely available cybertypes (μCT and montage images), might obviate the need for detailed written descriptions since all morphological information is visually available. This would alleviate one time consuming step, although the preparation of the cybertype adds additional time. However, there are valid arguments against this; notably that written descriptions are still necessary and should be part of taxonomic publications in the future. Even though readers from developed countries with fast internet can download publications with virtual imagery and access the raw data to examine and manipulate the cybertypes, this is not the case for scientists from developing countries with poor internet connectivity. With resources, as unevenly distributed as they are on a global scale, we propose to continue to have lengthy and detailed written descriptions, and at the same time also provide advanced images and 3D scans.

Despite the positive aspects of the application of μCT and cybertypes, there are also some weaknesses that deserve to be mentioned here. As already pointed out in previous studies [30, 42], access to μCT scanners is still very limited and often requires considerable costs for acquisition and/or usage, and will likely remain so for some time. An increasing number of natural history museums and universities are building up μCT facilities and providing access to them, but there is still a long way to go before a significant percentage of the taxonomic community will be able use the technology on a regular basis. Currently, it is also unclear if the community will embrace the new technology and how it will be used [30]. Furthermore, apart from access and costs to scanning resources, it has to be noted that handling the raw data, as well as post-processing it, requires time and technical skill. Another disadvantage is the sheer size of the data to be handled, which is more easily done with powerful (and expensive) computers and special databases for the curation of such data. Nevertheless, based on our own experiences and those documented in publications [12, 13, 30, 40, 42], we strongly believe that the advantages of the new technology outweigh the weaknesses. One should also consider that the application of μCT for systematics is relatively recent and the current disadvantages are natural for any emerging technology [30]. Technological improvements and a wider use will certainly reduce the costs of μCT and facilitate data processing.

#### From virtual to physical specimen models

**Fig 12:**
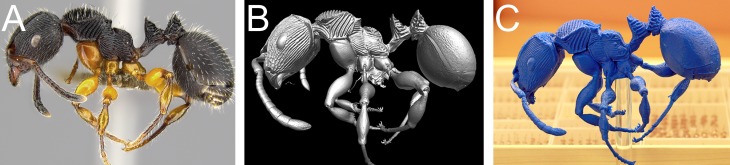
Different types of presentation of *T*. *balrog* sp. n. (CASENT0472559). Montage light microscopy image (A), virtual 3D model (B), 3D printed model (C).

MicroCT scans can also form the basis for a number of useful downstream products. One is the ability to print a physical model of the specimen in 3D, which can be used for a variety of research, educational, and outreach purposes. Single body parts or whole specimens can be printed and compared with other species without having to deal with physical specimens. Once enough volumetric datasets or cybertypes become available 3D prints generated from them can be used for more comprehensive comparative morphology studies and/or educational classroom purposes [78]. In addition, 3D prints can become valuable additions to real specimens in natural history collections, especially in cases where the physical specimen is difficult to examine, as for example fossil insects in amber [79]. In order to test this approach, we generated a 3D printed model of *T*. *balrog* (Fig 12). Such 3D printed specimens can also be used for public outreach in natural history museums or universities since it is often challenging to display small invertebrates to greater audiences. Visitors, students and the press can easily interact with 3D printed material without harm to the “real” specimens, and having a much larger model than the original minute-sized specimen gives a better impression of the animal’s morphology.

## Conclusions

Taxonomy is one of the oldest and most fundamental disciplines in biology. Taxonomic research seeks to organize information and build a documentary record of species. Every so often, technological advances change or enhance the practices and products of taxonomic science. In recent years, advances in informatics have enhanced taxonomic practice in numerous ways (e.g. electronic databases, online image repositories, etc.). Our study of two Malagasy *Terataner* species, along with other recent studies [44, 45, 77], demonstrate that μCT has much to offer the taxonomic community by allowing 3D quantitative information on organismal shape and morphology to exist in the digital realm. For ants, μCT combined with montage photos provides a nearly complete record of the phenotypic characters that are typically used in morphological taxonomy. In the near term, the widespread use of the technology will be limited due to lack of access to scanners and computational resources available to myrmecological communities around the world. But taxonomy is a long game, and these limitations will probably relax over time as technology advances and costs come down. Our study, and others [44, 45, 77], represent initial steps toward a 3D era for ant taxonomy.

## Supporting information

S1 Fig *Terataner balrog* sp. n. holotype worker (CASENT0472559). 3D PDF of volumetric surface model. (When viewing the 3D PDFs with Adobe Acrobat Reader (version 8 or higher), trusting the document by clicking on the image will activate the interactive 3D-mode and allows rotating, moving and zooming into the 3D model.)
(PDF)

S2 Fig *Terataner balrog* sp. n. paratype ergatoid queen (CASENT0426614). 3D PDF of volumetric surface model.
(PDF)

S3 Fig *Terataner nymeria* sp. n. holotype worker (CASENT0053630). 3D PDF of volumetric surface model.
(PDF)

S1 Video *Terataner balrog* sp. n. holotype worker (CASENT0472559). Volumetric surface rendering rotational video.
(AVI)

S2 Video *Terataner balrog* sp. n. paratype ergatoid queen (CASENT0426614). Volumetric surface rendering rotational video.
(AVI)

S3 Video *Terataner nymeria* sp. n. holotype worker (CASENT0053630). Volumetric surface rendering rotational video.
(AVI)
