# Supplementary figures and images for: X-Ray microtomography for ant taxonomy: An exploration and case study with two new Terataner (Hymenoptera, Formicidae, Myrmicinae) species from Madagascar
Source: PLoS One. 2017 Mar 22;12(3):e0172641. doi: 10.1371/journal.pone.0172641 (PMC5362212; doi:10.1371/journal.pone.0172641)

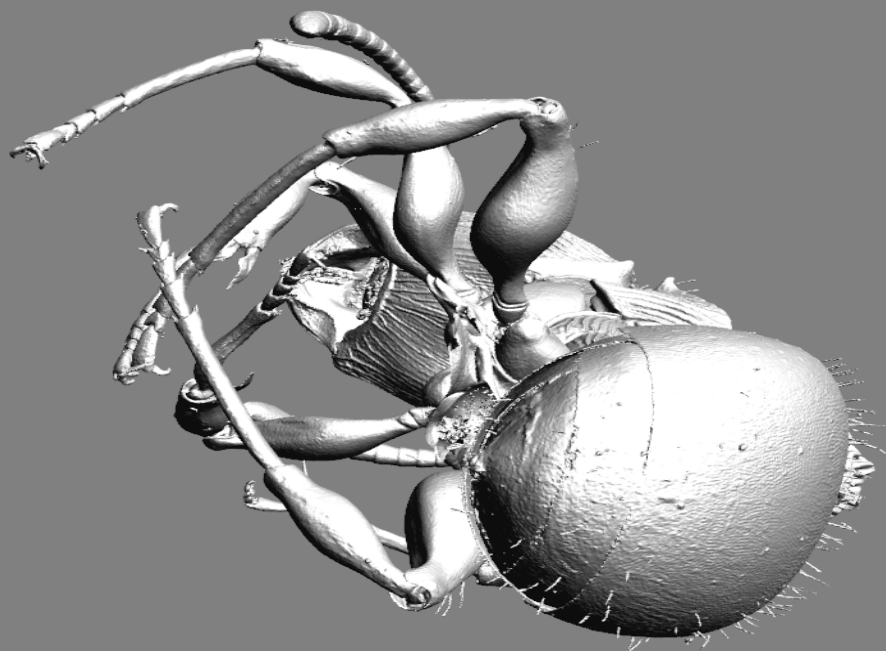

*Terataner balrog* (Holotype)  
Worker CASENT0472559

Supplement: S1 Fig — 3D PDF of volumetric surface model. (When viewing the 3D PDFs with Adobe Acrobat Reader (version 8 or higher), trusting the document by clicking on the image will activate the interactive 3D-mode and allows rotating, moving and zooming into the 3D model.) (PDF) [file pone.0172641.s001.pdf]

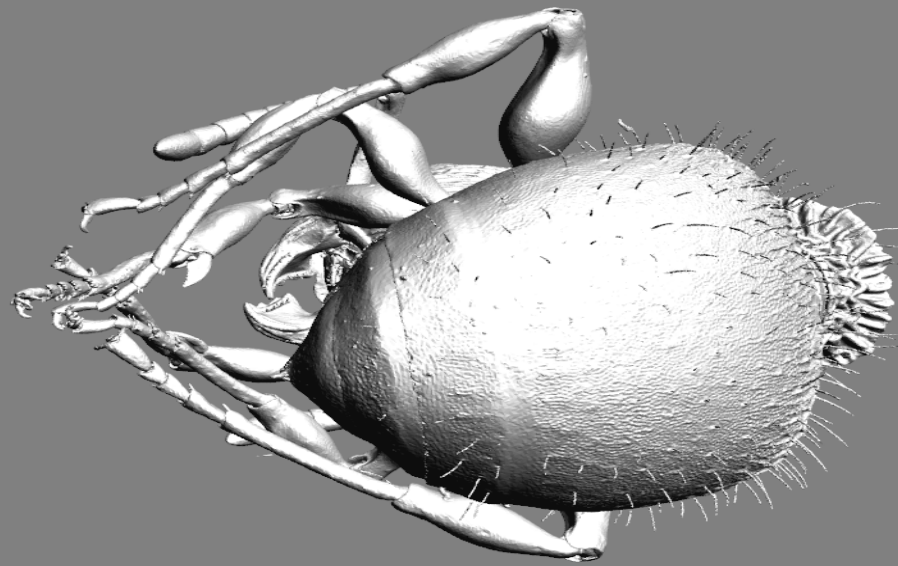

*Terataner balrog* (Paratype)  
Ergatoid queen CASENT0426614

Supplement: S2 Fig — 3D PDF of volumetric surface model. (PDF) [file pone.0172641.s002.pdf]

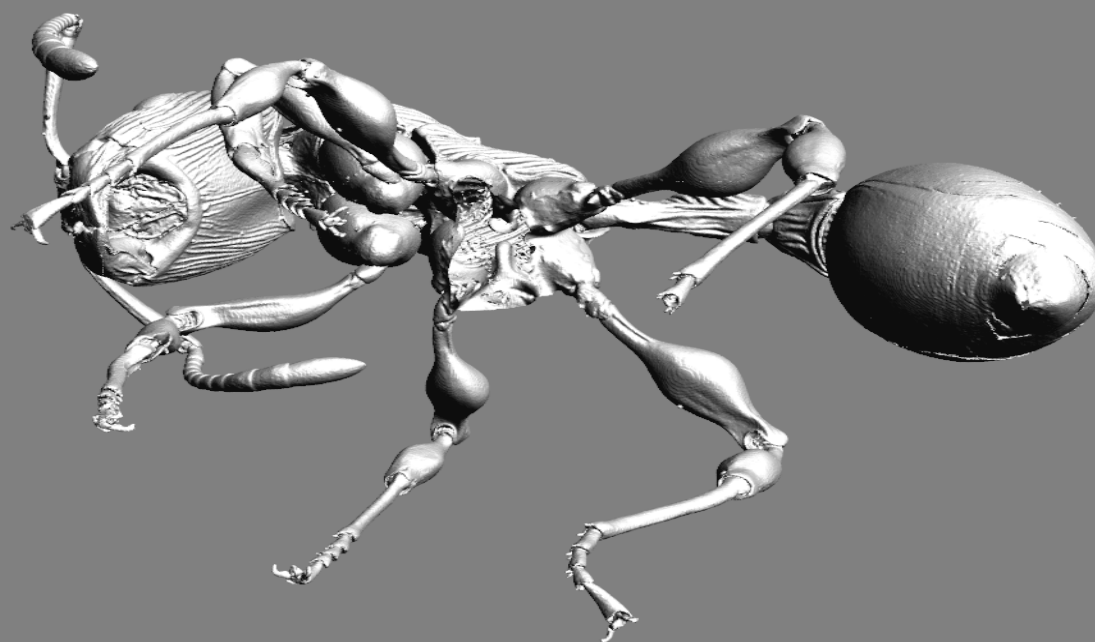

*Terataner nymeria* (Holotype)  
Worker CASENT0053630

Supplement: S3 Fig — 3D PDF of volumetric surface model. (PDF) [file pone.0172641.s003.pdf]
